# *Staphylococcus aureus* adapts to exploit collagen-derived proline during chronic infection

**DOI:** 10.1038/s41564-024-01769-9

**Published:** 2024-08-12

**Authors:** Andreacarola Urso, Ian R. Monk, Ying-Tsun Cheng, Camilla Predella, Tania Wong Fok Lung, Erin M. Theiller, Jack Boylan, Sofya Perelman, Swikrity U. Baskota, Ahmed M. Moustafa, Gaurav Lohia, Ian A. Lewis, Benjamin P. Howden, Timothy P. Stinear, Nicolino V. Dorrello, Victor Torres, Alice S. Prince

**Affiliations:** 1 Department of Pediatric Infectious Diseases, Columbia University, New York, NY USA; 2 Department of Pharmacology, Columbia University, New York, NY USA; 3 Department of Pediatrics, Columbia University, New York, NY USA; 4 Department of Microbiology and Immunology, University of Melbourne at the Peter Doherty Institute for Infection and Immunity, Melbourne, Victoria Australia; 5 Department of Pediatrics, Children’s Hospital of Philadelphia, Philadelphia, PA USA; 6 Department of Microbiology, New York University, New York, NY USA; 7 Department of Pathology, Columbia University, New York, NY USA; 8 Department of Biological Sciences, University of Calgary, Calgary, Alberta Canada; 9 Microbiological Diagnostic Unit Public Health Laboratory, The University of Melbourne at the Peter Doherty Institute for Infection and Immunity, Melbourne, Victoria Australia

**Keywords:** Infection, Bacteriology

## Abstract

*Staphylococcus aureus* is a pulmonary pathogen associated with substantial human morbidity and mortality. As vaccines targeting virulence determinants have failed to be protective in humans, other factors are likely involved in pathogenesis. Here we analysed transcriptomic responses of human clinical isolates of *S. aureus* from initial and chronic infections. We observed upregulated collagenase and proline transporter gene expression in chronic infection isolates. Metabolomics of bronchiolar lavage fluid and fibroblast infection, growth assays and analysis of bacterial mutant strains showed that airway fibroblasts produce collagen during *S. aureus* infection. Host-adapted bacteria upregulate collagenase, which degrades collagen and releases proline. *S. aureus* then imports proline, which fuels oxidative metabolism via the tricarboxylic acid cycle. Proline metabolism provides host-adapted *S. aureus* with a metabolic benefit enabling out-competition of non-adapted strains. These data suggest that clinical settings characterized by airway repair processes and fibrosis provide a milieu that promotes *S. aureus* adaptation and supports infection.

## Abstract (web summary)

*Staphylococcus aureus* upregulates collagenase and proline transporters to release collagen-derived proline and exploit fibroblasts as a nutrient source during chronic infection.

## Main

Among the numerous opportunistic bacteria that colonize the respiratory tract, *Staphylococcus* *aureus* is an especially successful pathogen, infecting both immunocompetent and immunocompromised hosts. While *S. aureus* can cause overwhelming necrotizing pneumonia^1^, it is most often associated with persistent pulmonary infections. As *S. aureus* is a major cause of healthcare-associated pneumonias, it is among the best-characterized human pathogens^2^. Its patterns of global epidemiology are well documented^3^, as are its abilities to regulate turnover of surface proteins^4,5^ and to secrete toxins^6,7^. Determinants of staphylococcal virulence have been extensively studied, and there is a wealth of data detailing its tissue-specific metabolic activity^8^. *S. aureus* has evolved to express an array of gene products that are specific to the human host, thwarting immune effectors, targeting surface receptors, blocking antibody and complement activity and activating excessive T-cell responses via superantigens^9,10^. Nonetheless, therapeutic and preventative strategies directed at several of these factors have been limited in their success to animal models of infection and have not been protective in human clinical trials^11,12^.

The pathogenesis of *S. aureus* pneumonia involves the aspiration of organisms from the upper respiratory tract and the establishment of a nidus of infection in the lower airways^7^. Airway damage, which may follow endotracheal intubation, viral infection or pulmonary diseases such as chronic obstructive pulmonary disease, idiopathic pulmonary fibrosis or cystic fibrosis (CF), is a major risk factor for *S. aureus* pulmonary infections^13^. Unfavourable outcomes are not limited to antibiotic-resistant strains. The bacterial mass, the recruited immune population and airway tissues contribute metabolites and oxidants at the site of infection^14^. Thus, a successful pathogen must adapt to the combined immune and metabolic pressures of the infected lung.

We postulated that *S. aureus* strains associated with persistent pulmonary infection acquire a substantial metabolic advantage driving their selection from the population of organisms that initially colonize the airways. We followed the transcriptional and metabolic changes in sequential clinical isolates from patients with pulmonary infection and determined that the successful *S. aureus* stains adapt to the lung by selectively consuming proline, generated by fibroblasts in the process of homeostatic collagen turnover and airway repair.

## Results

### Transcriptional changes in metabolic genes in vivo

**Fig. 1:**
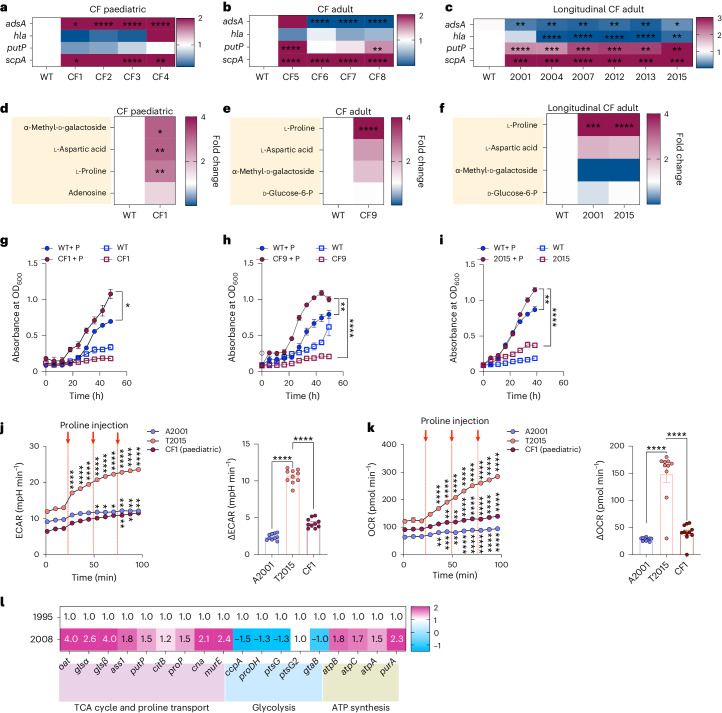
Patterns of gene expression in *S. aureus* clinical isolates. **a**–**c**, In vitro RNA transcripts from *S. aureus* CF clinical isolates grown in LB, all with respect to WT strain Newman control. Representative isolates CF1, CF9, 2001 and 2015 were chosen for functional studies. **d**–**f**, Utilization of carbon sources shown as fold change with respect to WT strain Newman control. ‘CF paediatric’ initial isolates (**a**,**d**) and ‘CF adult’ adapted isolates (**b**,**e**), from distinct patients; longitudinal CF adult adapted isolates (**c**,**f**) from single adult patient. **g**–**i**, Growth in CDM ± 100 μM proline. P, proline. Please note that (**g**), (**h**) and (**i**) refer to individual strains as labelled on the figure. **j**,**k**, Metabolic activity presented as ECAR (**j**) and OCR (**k**) upon sequential injections of 100 μM proline comparing initial paediatric (CF1) and later (A2001, T2015) isolates. mpH, milli-pH. **l**, RNA-seq data^19^ of early (1995) and late (2008) CF isolates from one patient over 13 years shown as fold change with respect to 1995. Data presented as mean ± s.e.m. In **a**–**k**, *n* = 3. Significance determined by *P < 0.05; **P < 0.01; ***P < 0.001 ****P < 0.0001. In **a**–**c**, one-way ANOVA with selective *t*-Student comparison; in **d**–**f**, two-tailed *t*-Student with Kolmogorov–Smirnov test comparing OCR increase upon proline injection; in **g**–**j**, two-way ANOVA with Dunnett’s multiple comparisons.

**Extended Data Fig. 1:**
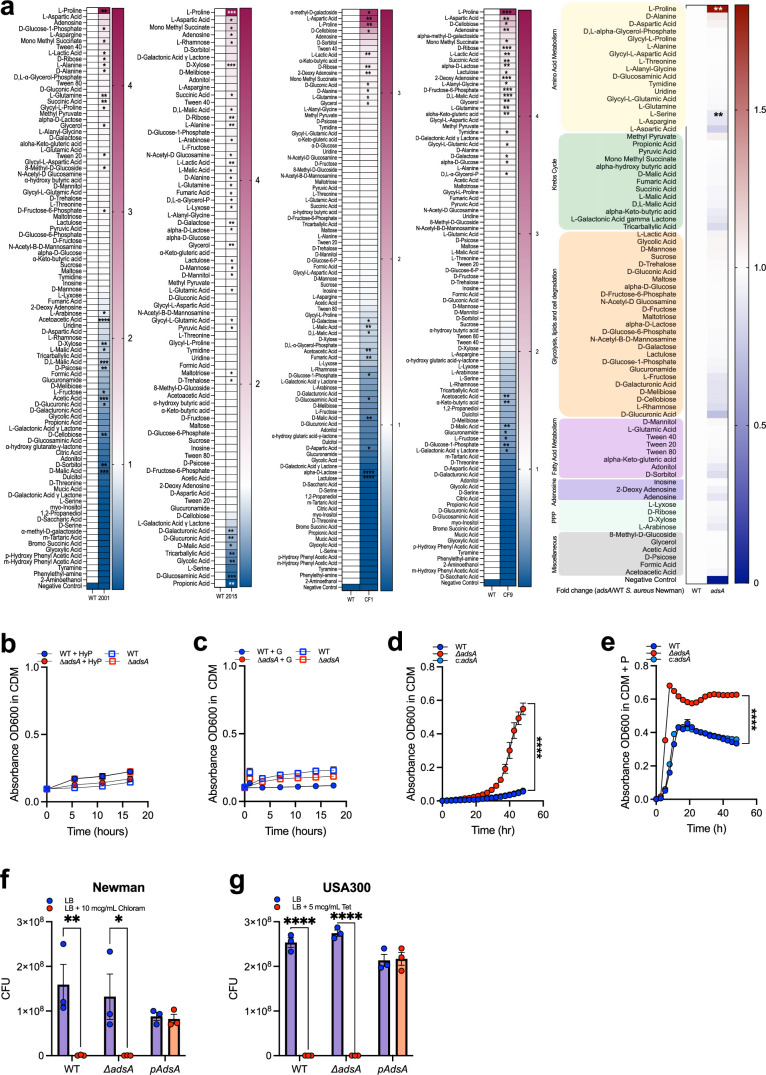
Relative single carbon source consumption by *S. aureus* strains. Utilization of carbon sources by CF clinical isolates (2001, 2015, CF1, CF9) and Newman *adsA* mutants shown as fold-change respective to Newman WT. **b**, Growth of *S. aureus* WT Newman and Δ*adsA* in CDM **b**, ± 100 μM hydroxyproline (HyP), **c**, ± 100 μM glycine (G), and c:adsA in **d**, CDM and **e**, CDM + proline. **f, g**, Newman and LAC plasmid stability in LB ± antibiotics. Mean ± SEM, n = 3. Significance determined by *P < 0.05; **P < 0.01; ***P < 0.001 ****P < 0.0001, (**a**) two-tailed t- Student with Kolmogorov-Smirnov test; (**b-e**) One-Way ANOVA with Tukey’s Multiple Comparisons; (**f, g**) Two- Way ANOVA with Dunnett’s Multiple Comparisons.

To determine how *S. aureus* adapts to the human lung following initial colonization, we surveyed the transcriptional profiles of initial clinical isolates from paediatric patients and chronic isolates from adult patients with CF. We targeted genes expected to be important in *S. aureus* pathogenesis such as the α-toxin *hla*, critical in initiating pulmonary infection^15^; *adsA*, which generates the potent immunosuppressant adenosine and thwarts immune clearance^16,17^; *putP*, one of the proline transporters activated in response to the availability of glucose^18^ which may be limited in the airways^19^; and *scpA*, a collagenase and protease^20^. Compared with a laboratory strain Newman (WT), *hla* expression was not increased in any of the isolates. The initial colonizing strains (non-adapted isolates labelled CF paediatric, 0.5–7 years of age) tended to have reduced *putP* and significantly elevated *adsA* expression compared with a laboratory control strain, whereas isolates from long-standing infection (adapted isolates labelled CF adult), instead had significantly reduced *adsA* but increased *putP* and *scpA*, with respect to a WT control (Fig. 1a,b). The isolates in a 15 year longitudinal collection of *S. aureus* from one adult patient (Longitudinal CF Adult) similarly had low *adsA* and high *putP* and *scpA* expression compared with WT control (Fig. 1c). While all isolates could utilize proline as a carbon source (Fig. 1d–f and Extended Data Fig. 1a), it was the most avidly consumed substrate by the adult isolates. Proline boosted the growth (Fig. 1g–i) and metabolic activity (Fig. 1j–k) of the adult isolates (T2015) to a significantly greater extent than the non-adapted paediatric strains. The T2015 strain also showed an increase in extracellular acidification rate (ECAR) compared with the paediatric initial strain or the 2001 isolate (Fig. 1j), but changes in ECAR were much lower than oxygen consumption rates (OCR) (Fig. 1k). These findings were consistent with RNA-sequencing data we mined from a study measuring differential gene expression in a late (2008) *S. aureus* clinical isolate^21^ versus early (1995) isolate from one CF patient, which also reflected upregulated tricarboxylic acid (TCA) cycle activity, proline transport and adenosine triphosphate (ATP) synthesis over 13 years of infection (Fig. 1l).

**Fig. 2:**
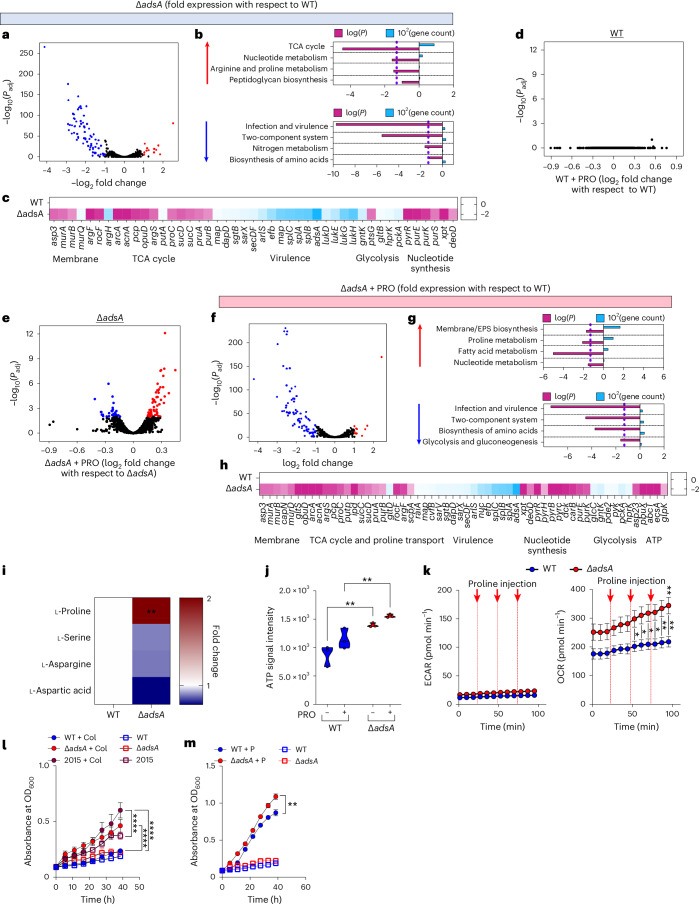
Metabolic activity of *adsA* mutant strain increases in response to proline in vitro. Bulk RNA sequencing of *S. aureus* WT and Δ*adsA* Newman grown in LB. **a**–**c**, Differential gene expression (**a**), pathway analysis (**b**) and fold change (**c**) in Δ*adsA* gene expression with respect to WT control. **d**,**e**, Differential gene expression of WT (**d**) and Δ*adsA* (**e**) in LB + 100 μM proline. **f**–**h**, Differential gene expression (**f**), pathway analysis (**g**) and fold changes (**h**) in Δ*adsA* genes with respect to WT control grown in LB + 100 μM proline. EPS, extracellular polysaccharide. **i**, Utilization of carbon sources shown as fold change with respect to WT control. **j**, ATP generated by WT control and Δ*adsA* strains grown in LB ± 100 μM proline. **k**, Carbon flux upon sequential injections of 100 μM proline in WT control and Δ*adsA* presented as ECAR and OCR. **l**,**m**, Growth of *S. aureus* WT control, Δ*adsA* and 2015 in ±30 μg ml^−1^ collagen (Col) (**l**) and growth of WT control and Δ*adsA* in ±100 μM proline (P) (**m**). Data graphics: in **a** and **d**–**f**, all dots represent individual genes; red dots, upregulated; blue dots, downregulated. In **b** and **g**, pink bars represent log(*P*); vertical dotted line is *P* < 0.05 threshold of significance; blue bars represent number of genes associated to the respective pathway. Data presented as mean ± s.e.m. In **a**–**h**, *n* = 2; in **i**–**m**, *n* = 3. Significance determined by *P < 0.05, **P < 0.01, ***P < 0.001, ****P < 0.0001. In **a**–**h**, Wald test generated *P* values (<0.05), and absolute log_2_ fold changes (>1*f* (greater than onefold)) were called differentially expressed; in **i**, **j**, **l** and **m**, two-way ANOVA with Dunnett’s multiple comparisons; in **k**, two-tailed *t*-Student with Kolmogorov–Smirnov test.

The patterns of transcription of both *scpA* and *putP* in clinical isolates seemed to be a logical response to a collagen and likely proline-rich milieu in the airway. Less obvious was the decreased expression of *adsA*, given its role in adenosine synthesis expected to promote infection and immune evasion^17,22^. We hypothesized that *adsA* expression must also have an impact on staphylococcal metabolism. We performed bulk RNA-sequencing on *S. aureus* Newman strains grown in Luria–Bertani (LB) media: a WT and an *adsA* mutant (Δ). Our results confirmed that loss of *adsA* caused global changes in gene expression (Fig. 2a–c). The *adsA* mutants significantly upregulated TCA cycle, nucleotide, arginine and proline metabolic pathways and downregulated amino acid biosynthesis and virulence gene expression with respect to the WT strain as established by pathway analyses (Fig. 2b). Specifically, loss of *adsA* increased expression of *arg*, *acnA* and *suc* loci and decreased expression of genes involved in glycolysis and virulence, including *ptsG*, *pckA*, *spl* and *luk* (Fig. 2c). We then assessed the effect of added proline, which induced no differential gene expression in the WT strain (Fig. 2d) but was associated with global changes in the Δ transcriptome (Fig. 2e). In response to proline, we observed significant shifts in Δ*adsA* metabolic pathways with respect to WT control (Fig. 2f), such as upregulated extracellular polysaccharide biosynthesis and proline metabolism itself (Fig. 2g), inducing expression of *putP*, *mur*, *asp*, *arg* and *scpA* while downregulating genes involved in glycolysis/gluconeogenesis and virulence, including *pckA*, *nuc*, *sar*, *spl*, *pde* and *pyk* (Fig. 2h). These genotypic effects were coupled to functional changes in which Δ*adsA* phenocopied the adapted clinical isolates in the utilization of proline as a primary carbon source (Fig. 2i and Extended Data Fig. 1a). ATP production by Δ*adsA* was increased compared with WT strain in LB media and further increased by the addition of proline (Fig. 2j), enhancing the OCR of Δ*adsA* but not WT strains (Fig. 2k).

Preferential proline consumption by adapted isolates suggested selection within the lung microenvironment for strains that could use collagen as a carbon source, a protein largely composed of proline, hydroxyproline and glycine^23,24^. While collagen and proline both increased Δ*adsA* and clinical strain 2015 growth (Figs. 1e and 2l,m), hydroxyproline and glycine did not (Extended Data Fig. 1b,c), indicating specificity in *S. aureus* carbon selection and the potential involvement of host collagen as a source of proline for bacterial metabolism. Overall, the metabolic profile of Δ*adsA* suggested that this mutant reflects the major genotypic and metabolic changes associated with the adaptation of the clinical isolates to the lung: the preferential consumption of proline and associated optimization of energy generation and growth.

### The airway metabolome provides proline for *S. aureus*

**Fig. 3:**
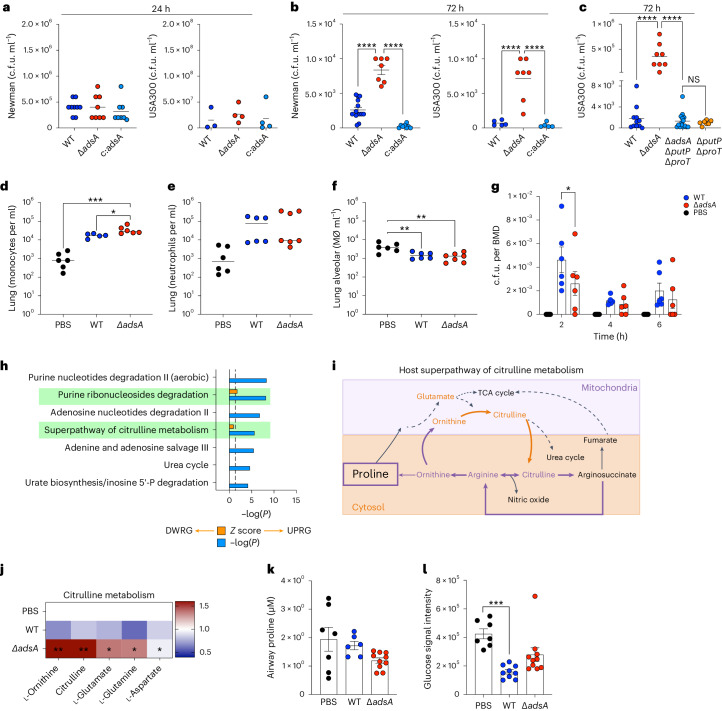
*S. aureus* infection induces citrulline metabolism in host airways. Pulmonary infection following intranasal inoculation with *S. aureus* WT Newman or USA300 LAC controls, Δ*adsA* or Δ*adsA-*complemented (c:*adsA*) strains. **a**,**b**, Lung bacterial burden at 24 h (**a**) and 72 h (**b**) post infection. **c**, Pulmonary infection following intranasal inoculation with *S. aureus* WT Newman or USA300 LAC controls, Δ*adsA* or Δ*adsA*Δ*putP*Δ*proT* strains. NS, not significant. **d**–**f**, Lung monocytes (**d**), neutrophils (**e**) and alveolar macrophages (**f**). **g**, Bacterial uptake and killing of murine BMDM assessed at 2, 4 and 6 h. **h**, Unbiased pathway analysis of BAL metabolites 72 h post-infection. Blue bars, *P* value; vertical dotted line is <0.05 threshold of significance; orange bars, *Z*-score or ratio of Δ*adsA* respective to WT; solid line is threshold of change. DWRG, downregulation; UPRG, upregulation. **i**, Schematic diagram of citrulline and proline metabolism in host; dotted arrows, translocation; solid arrows, conversion; in bold are critical metabolites/precursors to proline. **j**, BAL metabolites (selected from Supplementary Fig. 2a) related to citrulline metabolism, and targeted. **k**,**l**, Quantification of proline (**k**) and glucose (**l**) shown as fold change with respect to PBS. Data presented as mean ± s.e.m. from *n* = 3 in **a**; *n* = 4 in **b**–**f** and **h**–**l**; *n* = 2 in **g**. Significance determined by *P < 0.05, **P < 0.01, ***P < 0.001, ****P < 0.0001. In **a**–**f** and **j**–**l**, one-way ANOVA with Tukey’s multiple comparisons; in **g**, two-way ANOVA with Dunnett’s multiple comparisons; in **h**, two-tailed *t*-Student and fold change (*Z* > 0.5).

The in vivo selection of *S. aureus* strains that downregulate *adsA* expression and undergo a metabolic shift toward proline consumption and oxidative phosphorylation implies a survival advantage. We confirmed proline consumption to be higher in Δ*adsA* compared with both WT and complemented Δ*adsA* (c:Δ*adsA*) (Extended Data Fig. 1d,e), deemed to be a stable plasmid with or without antibiotics in both Newman and USA300 backgrounds (Extended Data Fig. 1f,g). Thus, we compared the recovery of WT, Δ*adsA* and c:Δ*adsA* in the Newman and USA300 backgrounds in acute (24 h) and sub-acute (72 h) mouse models of pneumonia. While the bacterial burdens were all comparable at 24 h post infection (Fig. 3a), there was significantly increased recovery of the Δ*adsA* in both the Newman and USA300 backgrounds at 72 h (Fig. 3b) compared with the WT or c:Δ*adsA*.

We confirmed the importance of proline, as opposed to other substrates that might become available to the organisms in the airway, by constructing an *adsA* mutant lacking the genes encoding both of the major proline transporters *putP* and *proT* in the USA300 background^25^. In the absence of *putP* and *proT*, the increased level of infection achieved by the *adsA* mutant was lost (Fig. 3c). Despite the published studies suggesting that *adsA* expression functions as a ‘virulence’ factor, we did not observe major changes in the recruitment of myeloid cells beyond the expected differences associated with bacterial load (Fig. 3d–f). Given the known role of AdsA in the inhibition of phagocytic killing, we evaluated possible differences in the susceptibility of the WT and *adsA* mutant to phagocytic clearance in vitro. In a gentamicin protection assay, the *adsA* mutant showed decreased survival within bone-marrow-derived macrophages (BMDM) (Fig. 3g). As might be predicted for the expected effects of *adsA* as a virulence factor^26^, we observed differences at 2 h following infection, where the *adsA* mutant was more readily killed, but by 4 h both WT and Δ*adsA* strains were killed equally. Given the lack of correlation of the in vitro myeloid cell functionality and the persistence of the *adsA* mutant in vivo, we postulated that the enhanced Δ*adsA* infection likely reflects a metabolic fitness advantage in the setting of established infection as preferred substrates become available.

**Extended Data Fig. 2:**
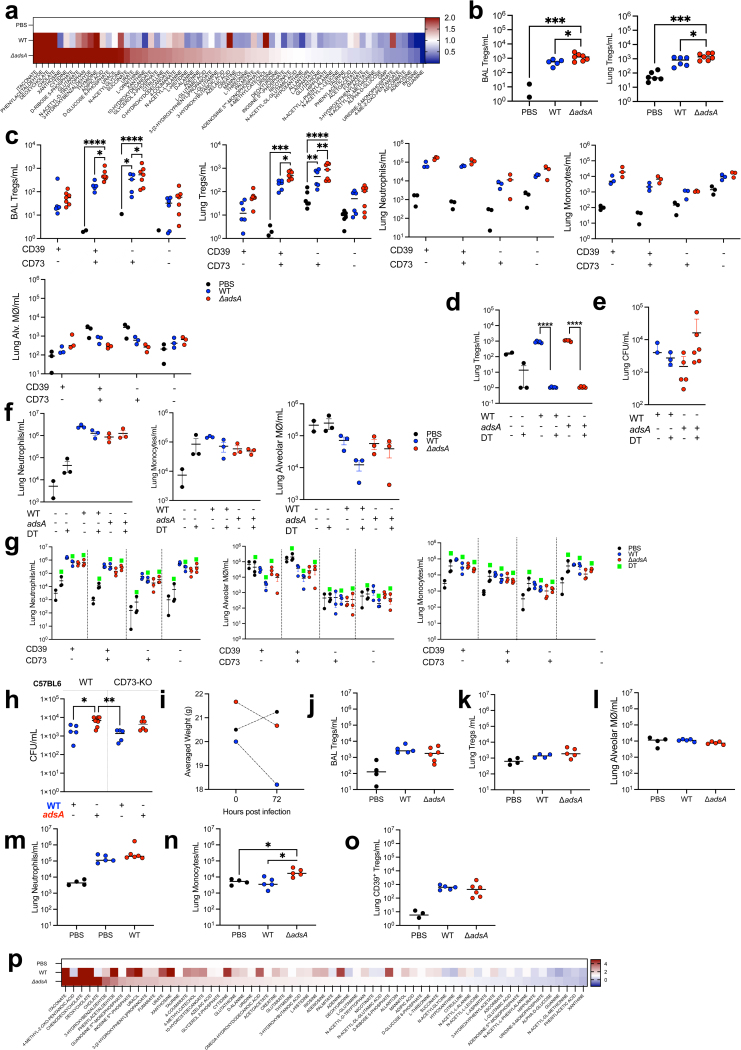
Impact of host generated adenosine on WT and Δ*adsA* mutant infection. Pulmonary infection at 72 hours following intranasal inoculation with *S. aureus* WT Newman, Δ*adsA* or PBS. **a**, Untargeted metabolomics shown respective to PBS control, **b**, T regulatory cell (Tregs) count and **c**, relative expression of surface CD39 and CD73 ectonucleotidases on various immune cells. Pulmonary infection at 72 hours following intranasal inoculation of GFP FoxP3^DTR^ mice with *S. aureus* WT Newman or Δ*adsA* and *in vivo* diphtheria toxin (DT)-mediated Treg depletion. **d**, Total lung Tregs **e**, bacterial burden **f**, immune cell populations and **g**, CD39 and CD73 expression upon treatment with DT or vehicle. Pulmonary infection at 72 hours following intranasal inoculation of CD73-KO mice with *S. aureus* WT Newman or Δ*adsA*. **h**, Bacterial burden in WT and CD73-KO mouse lungs. **i**, Weight loss, **j-o**, lung immune cells, and **p**, untargeted metabolomics on BAL fluid respective to PBS. Mean ± SEM, (**a, b**) n = 3; (**c-e, h-p**) n = 3; (**f**) n = 4. Significance determined by *P < 0.05; **P < 0.01; ***P < 0.001 ****P < 0.0001; (**a-h. j-p**) One- Way ANOVA with Tukey’s Multiple Comparisons; (**f**). Two-Way ANOVA with Dunnett’s Multiple Comparisons.

To better understand the metabolic impact of *adsA* expression on *S. aureus* persistence in the lung, we performed a metabolomic study on mouse bronchoalveolar lavage (BAL) fluid collected at 72 h of infection with the WT or Δ*adsA* (Extended Data Fig. 2a). Non-biased pathway analyses indicated a significant upregulation of both purine degradation and citrulline metabolism in Δ*adsA* infected airways (Fig. 3h).

We evaluated the impact of host purine degradation as an explanation for the enhanced Δ*adsA* survival in vivo, compared with the WT strain. Host adenosine is predominantly generated through the sequential activity of CD39 (cluster of differentiation 39) (adenosine diphosphate to adenosine monophosphate (AMP)) and CD73 (AMP to adenosine) nucleotidases^27^ expressed by regulatory T (T_reg_) cells. We found that T_reg_ cells from Δ*adsA*-infected mice expressed more ectonucleotidase than those infected with the WT control (Extended Data Fig. 2b,c), possibly compensating for the total pool of adenosine. However, neither T_reg_ cell depletion nor global elimination of CD73 (Extended Data Fig. 2i,j) altered bacterial recovery from the lung (Extended Data Fig. 2d–f), indicating that host adenosine production is not responsible for the increased survival of Δ*adsA mutant* in the lung.

**Extended Data Fig. 3:**
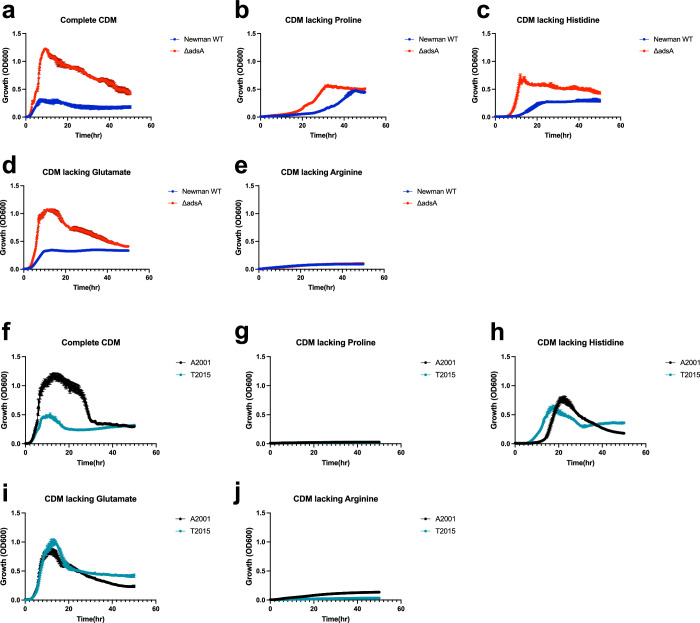
Consumption of components of the citrulline superpathway by ∆*adsA* and CF clinical isolates. Growth of **a-e**, WT, Δ*adsA*, **f-j**, A2001 and T2015 in **a, f**, complete defined medium (CDM) lacking **b, g**, proline, **c, h**, histidine, **d, i**, glutamate or **e, j**, arginine. Mean ± SEM from (**a-j**) n = 4.

We proceeded with evaluating the superpathway of citrulline metabolism (Fig. 3i) as the basis for selective advantage of *S. aureus* strains with decreased *adsA* expression in vivo. The accumulation of ornithine, citrulline, glutamate and glutamine in the Δ*adsA* infected airway were consistent with an increase in proline metabolism (Fig. 3j). However, given the involvement of proline in a network of metabolic pathways characterized by the abundance of several other amino acids, we further assessed the central role of proline as the preferred substrate for the Δ*adsA* and clinical host-adapted strains in vivo. We compared growth in defined media supplemented with other amino acids that generate glutamate (Extended Data Fig. 3a–e). In the absence of proline, growth of WT and Δ*adsA* were both impaired; the addition of histidine or arginine, but not glutamate, enhanced growth of the Δ*adsA* but not the WT parental strain. The CF clinical isolates had an absolute requirement for added proline or arginine (Extended Data Fig. 3f–j). Strain T2015 utilized these amino acids in vitro to varying extents, whereas neither A2001 nor T2015 could utilize arginine. Thus, in vitro, both the Δ*adsA* and the clinical isolates are able to exploit other available amino acids that are provided exogenously, and their enhanced growth is not necessarily limited to proline uptake, as was preferred in vivo.

Targeted metabolomics indicated a trend toward decreased proline in Δ*adsA* infected airways (Fig. 3k) and a significant decrease in glucose (Fig. 3l), usually a preferred *S. aureus* substrate but only associated with the WT strain compared with a phosphate-buffered saline (PBS) control. These findings overall are consistent with a metabolic switch by the Δ*adsA* mutant and the clinical strains to proline catabolism in the setting of established infection, as *S. aureus* prefers to transport and not synthesize proline when glucose is perceived as limited^18,25,28^.

**Fig. 4:**
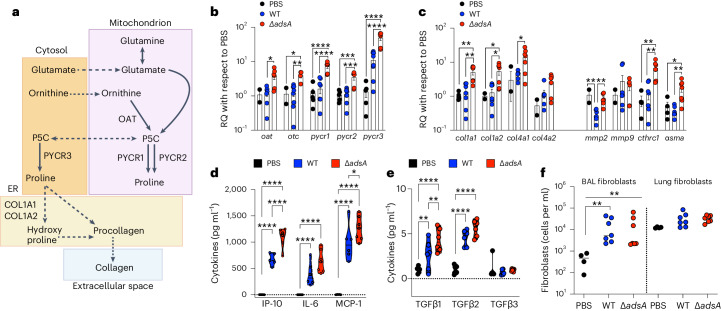
*S. aureus* activate genes involved in collagen synthesis. **a**, Schematic diagram of proline and collagen biosynthesis pathways in the host. Dotted arrows, translocation; solid arrows, conversion; in bold are critical enzymes. **b**,**c**, At 72 h following intranasal inoculation with *S. aureus* Newman WT control or Δ*adsA*, we monitored host lung RNA transcripts associated with proline biosynthesis (**b**) and collagen biosynthesis turnover via *mmp*s and markers of fibroblast activity (**c**). **d**,**e**, Selected cytokines were quantified in BAL. **f**, CD140a^+^SCA-1^+^ fibroblast numbers in lung and BAL fibroblasts quantified. Data presented as mean ± s.e.m. In **b**–**f**, *n* = 3. Significance determined by *P < 0.05, **P < 0.01, ***P < 0.001, ****P < 0.0001. In **b**–**f**, one-way ANOVA with Tukey’s multiple comparisons.

**Extended Data Fig. 4:**
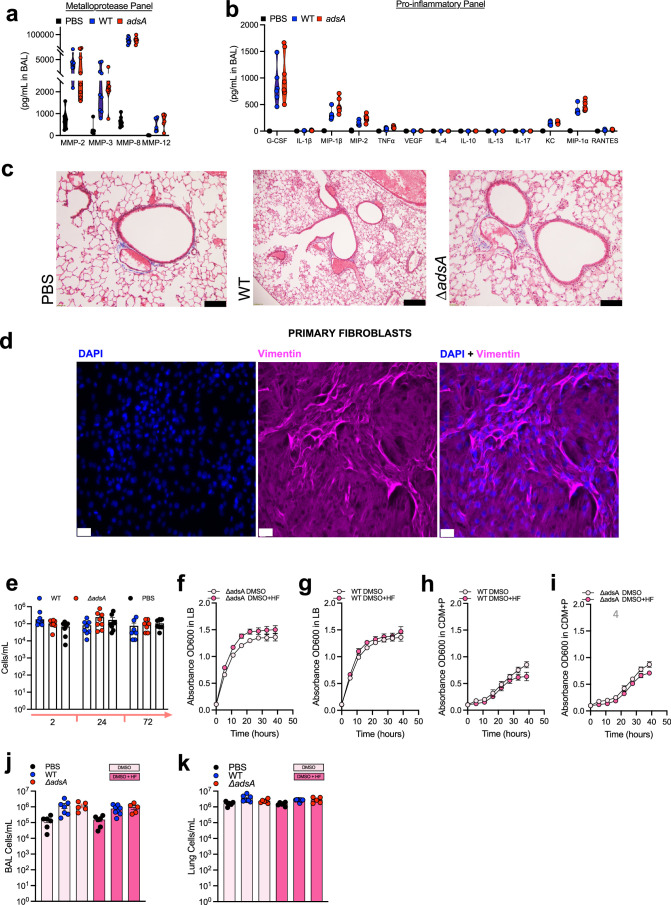
Induction of collagen synthesis by WT and ∆*ads*A infection. Induction of host responses by pulmonary infection with *S. aureus* WT or Δ*adsA* strains at 72 h. **a**, BAL fluid matrix metalloprotease and **b**, pro-inflammatory cytokines quantified by ELISA and plotted with respect to PBS. Primary murine fibroblasts were freshly isolated and cultured for infection with *S. aureus* WT or Δ*adsA* strains and treatment with collagen inhibitor halofuginone (HF) ex vivo. **c**, Representative lung sections stained with Masson’s Trichrome (purple) and hematoxylin and eosin staining (pink) from a PBS control, WT Newman and Δ*adsA* infection at 72 h are shown. Scale bar= 50 μM; m = 40x. **d**, Representative confocal images of primary fibroblasts (PF) stained with vimentin and DAPI to confirm population purity (scale bar 40μm). **e**, Cell count of live PF with trypan blue at each infection timepoint *in vitro*. *S. aureus* growth in **f, g**, LB only and **h, i**, CDM + P supplemented with HF or vehicle. *In vivo* intranasal 72- hour lung infection with *S. aureus* WT or Δ*adsA* strains in mice treated with HF or vehicle. **j**, BAL and **k**, lung total cells from infected mice treated with HF or vehicle. Mean ± SEM from (**a-b**) n = 3; (**e**) n = 4; (**f-i**) n = 6; (**j-k**) n = 6. (**a-b, d-j**) Two-Way ANOVA with Dunnet’s Multiple Comparison.

While BAL represents a combination of eukaryotic and prokaryotic products, we expected most of these metabolites to be of eukaryotic origin. We verified the eukaryotic origin of the staphylococcal substrates by demonstrating the upregulation of the corresponding host enzymes involved in proline and collagen (I and IV) synthesis (Fig. 4a) and greater abundance in Δ*adsA* infected hosts (Fig. 4b,c). Increased transcription of collagen triple-helix repeat complex (*cthrc*) and alpha smooth muscle actin (*α-sma*) suggested the participation of activated fibroblasts in Δ*adsA* infection (Fig. 4c) as did the combination of increased IP-10, IL-6, MCP-1 and TGFB1 (Fig. 4d,e)^29,30^. Despite the increased expression of fibroblast-associated genes in Δ*adsA* infection (Fig. 4a–c), fibroblast numbers in either BAL or lung were equivalent to WT (Fig. 4f). We also explored other mechanisms of collagen turnover which can foster a similar response^31^. We found roughly equivalent production of matrix metalloproteases (MMPs) in BAL (Extended Data Fig. 4a) and their transcripts (*mmp*) in the lung (Fig. 4c) which are typically activated in frank tissue damage (Extended Data Fig. 4a). Cytokines and chemokines commonly associated with stress and inflammation during infection were also comparable in Δ*adsA* and WT infected hosts (Extended Data Fig. 4b), as was the gross histology of the infected lung (Extended Data Fig. 4c). These findings suggest that this host response was specific to Δ*adsA* infection and not simply a reflection of the increased Δ*adsA* bacterial burden or frank tissue damage. Thus, Δ*adsA* mutants appear to enjoy an advantage in vivo by virtue of their ability to exploit available proline, which seems likely to be synthesized by activated fibroblasts.

### Airway fibroblasts provide proline for *S. aureus* consumption

**Fig. 5:**
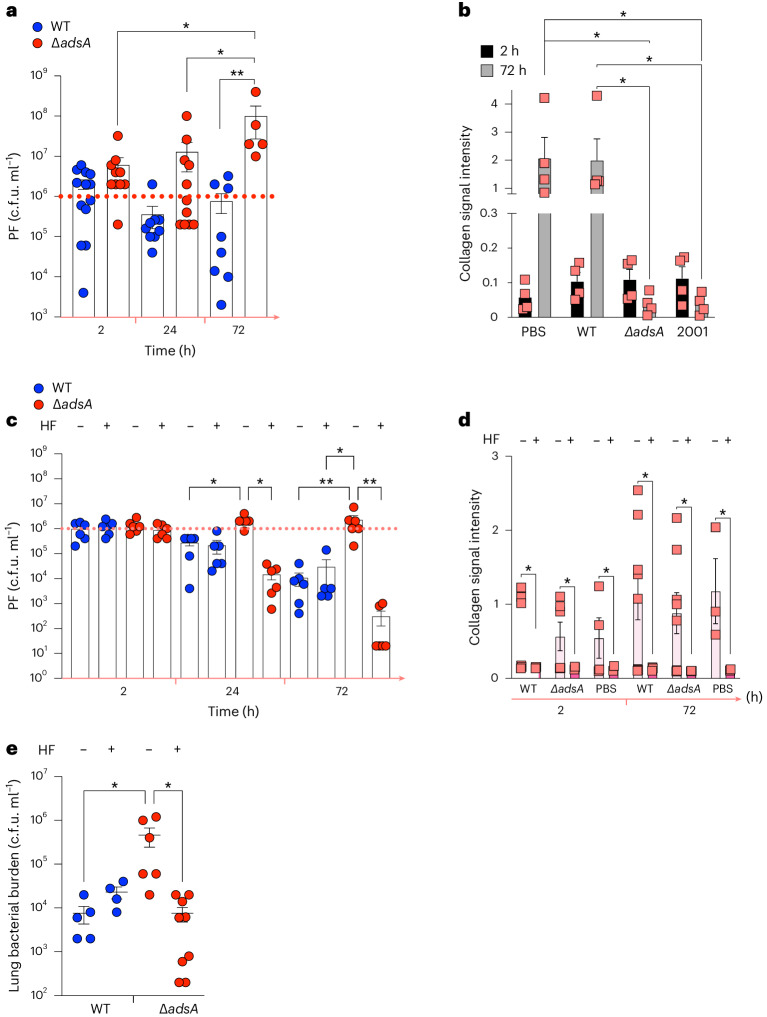
Inhibition of collagen synthesis suppresses *S. aureus* growth. Primary murine fibroblasts were infected with *S. aureus* WT control or Δ*adsA* strains. **a**,**b**, Bacterial burden (**a**) and quantification of collagen by ELISA (**b**) in primary murine fibroblasts culture supernatants following infection with WT control, Δ*adsA* or clinical strains. **c**,**d**, Bacterial burden (**c**) and collagen quantification by ELISA (**d**) in primary murine fibroblast cultures treated with halofuginone, an inhibitor of collagen synthesis or vehicle (DMSO). **e**, Bacterial burden 72 h post-WT control or *ΔadsA* infection of mice treated with intranasal halofuginone or DMSO. Data presented as mean ± s.e.m. In **a**–**d**, *n* = 6; in **e**, *n* = 3. Significance determined by *P < 0.05, **P < 0.01. In **a**–**d**, two-way ANOVA with Brown–Forsythe test; in **e**, one-way ANOVA with Tukey’s multiple comparisons. PF, primary murine fibroblasts; HF, halofuginone.

We sought to confirm the role of fibroblasts as the source of collagen and hence proline fuelling Δ*adsA* growth. We isolated primary murine fibroblasts (Extended Data Fig. 4d) to use as a substrate for *S. aureus* infection. We observed that while the WT bacterial burden remained unchanged over time, the Δ*adsA* bacterial burden was significantly increased at the 72 h time point (Fig. 5a and Extended Data Fig. 4e), coincident with the depletion of collagen in the fibroblast cultures infected with either Δ*adsA* or the 2001 clinical isolate, but not the WT or PBS control at 72 h (Fig. 5b). We inhibited collagen synthesis by the fibroblasts with halofuginone^32–34^, which significantly decreased the Δ*adsA* bacterial burden but not that of WT controls and corresponded with collagen depletion in the culture supernatant (Fig. 5c,d**)**. As in the untreated condition, we observed increased Δ*adsA* bacterial burden compared with WT at 24 and 48 h post infection. We confirmed the in vivo significance of ongoing collagen synthesis in selectively supporting infection by the Δ*adsA* mutants as Δ*adsA* mice treated with halofuginone (by the intraperitoneal route) lost their in vivo advantage. Infection by WT *S. aureus* at 72 h was not significantly altered by halofuginone compared with a vehicle control (Fig. 5e). Neither bacterial (Extended Data Fig. 4f–i) nor fibroblast (Extended Data Fig. 4j,k) viability was significantly decreased by halofuginone. This set of experiments strongly supports our hypothesis that host-adapted clinical isolates of *S. aureus*, as well as our surrogate Δ*adsA* strain with similar metabolic properties, exploit host fibroblast deposition of collagen and hence proline synthesis to persist in the lung.

### Carbon catabolite repression directs proline utilization

**Fig. 6:**
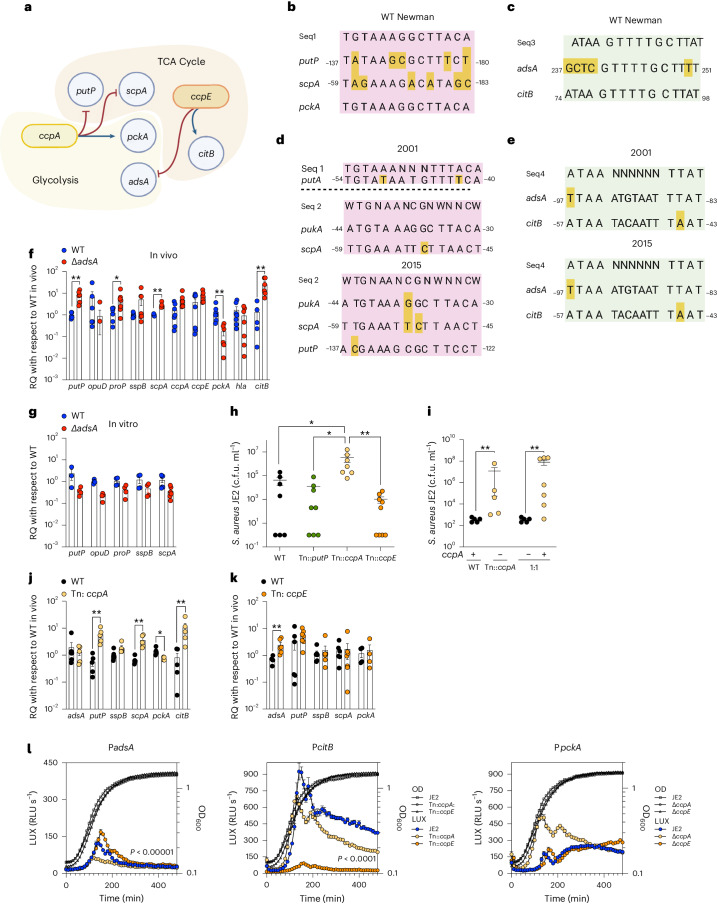
CCR regulates *S. aureus* metabolism in the lung. **a**, Schematic diagram of proposed CCR regulation of *S. aureus* consumption of collagen and proline. **b**–**e**, In silico identification of CRE binding sites using composite and verified consensus sequence targets for CcpA (Seq1, Seq2), known to regulate *pckA*, and CcpE (Seq3), known to regulate *citB*. CcpA homologous sequences in pink; CcpE homologous sequences in green. Putative consensus sequences in *S. aureus* Newman WT (accession number NC_009641.1) indicating CcpA regulation of *putP*, *scpA* and *pckA* (control) (**b**) and CcpE regulation of *adsA* and *citB* (control) (**c**); yellow highlighting, mismatch. Putative consensus sequences in *S. aureus* respiratory clinical isolates 2001 and 2015 indicating CcpA regulation of *putP*, *scpA* and *pckA* (control) (**d**) and CcpE regulation of *adsA* and *citB* (control) (**e**); yellow highlighting, mismatch. **f**–**k**, Effects of CcpA and CcpE in mice infected with *S. aureus* WT Newman control and Δ*adsA* (**f**,**g**) or JE2 WT control and isogenic *ccpA*::Tn, *ccpE*::Tn and *putP*::Tn transposon mutants (**h**–**k**) for collection of prokaryotic RNA and bacterial burden at 72 h. **f**,**g**, Transcripts from in vivo lungs (**f**) and respective in vitro inoculum (**g**) grown in LB. **h**,**i**, Lung bacterial burden (**h**) and bacterial in vivo competition (**i**). **j**,**k**, In vivo transcripts from *ccpA*::Tn (**j**) or *ccpE*::Tn (**k**) compared with a WT JE2 control in the lung. **l**, Activation of the *adsA* promoter detected using a LUX reporter expressed in *ccpA*::Tn, *ccpE*::Tn or JE2, shown as a function of bacterial growth (OD_600_). Data presented as mean ± s.e.m. In **f**, *n* = 4; in **g** and **l**, *n* = 3; in **h**, *n* = 4; in **i**, *n* = 5; in **j** and **k**, *n* = 4. Significance determined by *P* < 0.05, **P < 0.01, ***P < 0.001, ****P < 0.0001. In **f**, **g**, **j** and **k**, two-tailed multiple *t*-Student with Kolmogorov–Smirnov tests; in **h**, one-way ANOVA with Kruskal–Wallis comparisons; in **i**, Mann–Whitney test; in **l**, two-way ANOVA with Dunnett’s multiple comparisons.

Our data thus far suggested that *S. aureus* clinical isolates, along with Δ*adsA* mutants that also consume host-generated proline, enjoy a growth advantage in vivo compared with WT organisms. In vitro, carbon catabolite repression (CCR) directs the *S. aureus* metabolic switch from glycolysis, fuelled by glucose, to proline metabolism and oxidative phosphorylation via the TCA cycle^31,35^. The perception of limited glucose in the airway is mediated by carbon catabolite protein A (CcpA) and phosphorylated Hrp to direct the expression of >300 genes^36,37^, a complex system conserved in many bacterial species^38–40^. CCR also provides a mechanism to link bacterial metabolism and the expression of virulence determinants^41,42^. In *S. aureus*, CcpA negatively regulates *putP* and other amino acid transporters and positively regulates *pckA*, a gene central to glycolysis^35^, whereas carbon catabolite protein E (CcpE) positively regulates *citB* and feeds the TCA cycle activity^43^ (Fig. 6a). It seemed likely that CCR is the dominant factor regulating *S. aureus* metabolism in the lung and drives selection of the host-adapted clinical isolates as well as the parallel metabolic behaviour of the *adsA* mutant.

We utilized a bioinformatic approach to verify the presence of catabolite responsive elements (CRE) associated with both CcpA and CcpE in the promoter regions of selected genes in the clinical isolates 2001 and 2015 (Supplementary Tables 2 and 3) and in the Newman genome. Published consensus sequences associated with CcpA regulation (TGTAAAANNNTTTACA, WTGNAANCGNWNNCW)^44,45^ were found in *putP* and *pckA* as expected^35,46^ and also in the promoter region of the collagenase *scpA* (Fig. 6b,d) in Newman strains and 2001 and 2015 isolates. A composite sequence associated with CcpE regulation of *citB* (ATAANNNNNNTTAT)^47^ was identified in both of these clinical isolates and Newman strains in the promoter region of *adsA* (Fig. 6c,e).

We predicted that *adsA* must also be regulated by CCR which would explain the preferential consumption and growth on proline in vivo as well as reliance on oxidative phosphorylation and the TCA cycle to generate ATP. We compared rates of transcription of our selected genes in organisms collected directly from the murine lung at 72 h of infection with WT or Δ*adsA* compared with bacteria grown in LB (Fig. 6g). The Δ*adsA* strains had increased *putP*, *scpA* and *citB* but decreased *pckA* expression (Fig. 6f), consistent with positive regulation by CcpE activity and with the inactivity of CcpA.

The impact of CCR regulation on *S. aureus* in vivo was addressed by testing the ability of mutants lacking specific CCR elements to cause pneumonia. Bacterial burden in mice infected with *ccpA*::Tn in the JE2 background stain was greater than that of mice infected with WT, *ccpE*::Tn or *putP*::Tn strains (Fig. 6h). A competition experiment infecting mice with equal inocula of the WT and *ccpA*::Tn strains also illustrated the advantage of the *ccpA* mutant (Fig. 6i) also indicating that the loss of *adsA* expression increases persistence in the lung. We noted increased expression of *putP*, *scpA* and *citB* but decreased expression of *pckA* in *ccpA*::Tn infection, compared with WT control (Fig. 6j). *ccpE*::Tn *S. aureus* collected directly from infected mice showed increased expression of *adsA* and achieved similar levels of infection as the WT strain (Fig. 6k,h). The combination of inactive CcpA with active CcpE generates the pattern of gene expression observed in vivo in the Δ*adsA* and adapted clinical isolates (Figs. 1b,c and 6f).

We confirmed CCR regulation of *adsA* expression using a luciferase (LUX)-promoter fusion construct to monitor the relative activation of the *adsA* promoter in the parental JE2 background, *ccpA*::Tn or *ccpE*::Tn strains, having established equivalent rates of growth over the assay conditions (Fig. 6l). In the *ccpA*::Tn strain, we observed less *adsA* reporter activity, compared with the *ccpE-*deficient strain in which *adsA* expression was significantly increased (Fig. 6l). The control for our promoter fusion assay documented the expected positive regulation of *citB* by CcpE and the relative lack of influence of CcpA on its expression^43^. The CCR regulatory system, which optimizes staphylococcal bioenergetics, directs *S. aureus* metabolism in this setting in which collagen deposition, as part of the ongoing process of host airway repair, provides proline.

**Extended Data Fig. 5:**
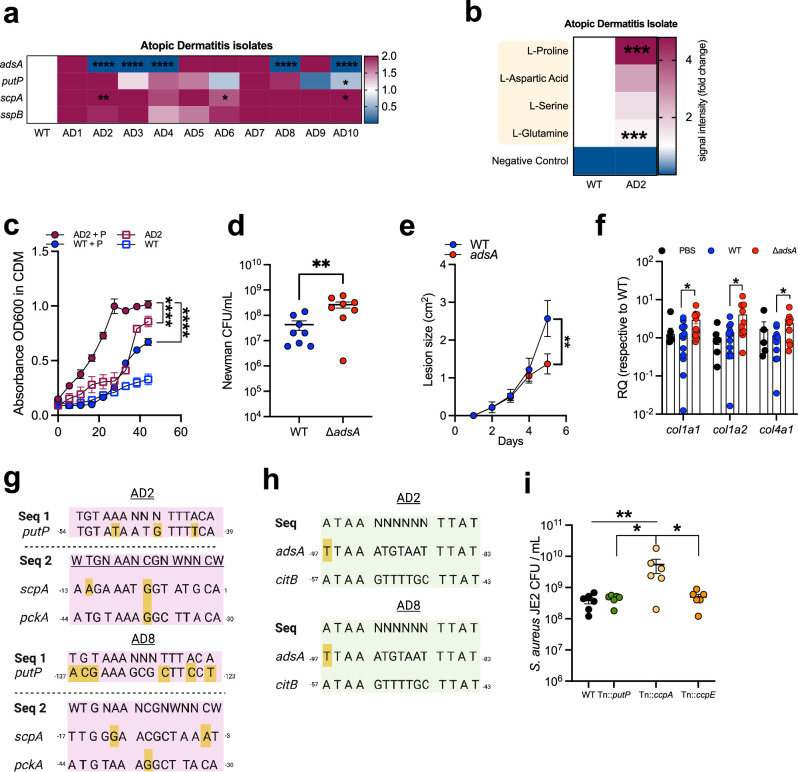
Impact of *adsA* and CCR on skin infection. **a**, RNA transcripts from *S. aureus* Atopic Dermatitis (AD) clinical isolates. **b**, Carbon utilization assays shown as fold-change increase w.r.t. WT. **c**, Growth curves in CDM ± 100 μM proline. **d**, Bacterial burden and **e**, skin lesion size at 6 days post-infection from a 5 mm punch biopsy of skin infected with *S. aureus* Newman. **f**, Host RNA transcripts from 5 mm punch biopsy of skin infected with *S. aureus* Newman. **g-h**, Putative *cre* binding sites in *S. aureus* atopic dermatitis (AD) isolates AD2 and AD8 for *ccpA* in silico (black dashes, mismatches; red dots, matches) using binding sequences Sequence 1 and Sequence 2 (red). **i**, Bacterial burden at 6 days post- infection from a 5 mm punch biopsy of skin infected with WT JE2, *ccpA::*Tn*, ccpE::*Tn*, putP::*Tn. Data presented as mean ± SEM, (**a-c**) *n* = 3, (**d,e**) *n* = 4, (**i**) *n* = *3*. Significance determined by *P < 0.05, **P < 0.01, ***P < 0.001, ****P < 0.0001, (**a,c,e**) Two-Way ANOVA with Dunnett’s Multiple Comparisons, (**b,d**) two-tailed t- Student with Kolmogrov-Smirnov test (**f,i**) One-Way. ANOVA with Tukey’s Multiple Comparisons.

CCR is engaged in *S. aureus* adaptation to available nutrients, and hence, we predicted that isolates that had undergone adaptation to another collagen-rich site, such as the skin, would also show CCR-directed metabolic activity. We screened ten *S. aureus* strains from children with atopic dermatitis, although the chronicity of infection was not known, for patterns of gene expression and preferred substrate utilization (Extended Data Fig. 5a). Roughly half of the isolates reflected CCR control with suppressed *adsA* and increased *putP* and *scpA* expression. A representative strain AD2 showed significant preference for proline consumption compared with a strain Newman control and showed increased growth rates in proline (Extended Data Fig. 5b,c). Using the Δ*adsA* mutant as a surrogate for the clinical and less virulent host-adapted strains, we observed increased ability to infect murine skin (Extended Data Fig. 5d) generating smaller lesion sizes compared with WT (Extended Data Fig. 5e). We also found increased induction of host collagen synthesis, with increased expression of *col1a*1, c*ol1a2* and *col4a1* (Extended Data Fig. 5f), findings similar to those of the pulmonary isolates. We utilized the same bioinformatic approach to verify the presence of CRE associated with both representative clinical isolates AD2 and AD8 in the promoter regions of selected genes and found similar matches (Extended Data Fig. 5g,h). We also found that the *ccpA::Tn* mutant was associated with significantly greater bacterial loads in a murine skin infection model, consistent with the pattern of increased infection in the lung (Extended Data Fig. 5i). Thus, CCR direct gene expression in *S. aureus* isolates to optimize utilization of available carbon sources is not limited to the lung.

Our findings overall indicate that *S. aureus* adapts its metabolic activity in the lung and perhaps at other sites as directed by CCR. In a setting of accessible and abundant collagen, as in the airway and possibly the skin, the suppression of CcpA activity enables utilization of amino acid transporters. The organisms that preferentially utilize proline derived from collagen show a substantial growth advantage and establish persistent infection.

## Discussion

*S. aureus* is a complex pathogen that optimizes its metabolic activity according to the constraints of the local environment. We found that organisms that persist in the lung exploit the availability of proline released from collagen, which is actively synthesized by fibroblasts during the process of tissue repair. Proline consumption along with expression of *adsA* is responsible for the increased growth of clinical isolates in the damaged lung. These metabolic properties apparently predominate over the expected anti-inflammatory consequence of AdsA as have been described in models of sepsis or renal abscess formation^48–50^ and indicate tissue-specific adaptation. Strategies to prevent or suppress *S. aureus* infection have focused upon their expression of multiple toxins and gene products that interfere with host immune clearance^6–8^. While these genes may be important in the initial stages of infection, an increasing literature indicates that the organisms causing more persistent infection have an entirely different pattern of gene expression. Such host-adapted strains have substantially altered metabolic activity, giving them a growth advantage specifically in the local microenvironment. Our data were generated from *S. aureus* strains isolated over years of chronic infection compared with initial isolates or laboratory strains. Exactly how long it takes for *S. aureus* metabolic rewiring to take place in vivo remains to be established and whether analogous metabolic changes occur in S*. aureus* at other sites of infection remains to be established. Appreciating the metabolic directives that drive *S. aureus* gene expression in vivo may provide novel targets for therapy or infection prevention in patients, especially those with damaged airways, fibrosis and collagen deposition who are at increased risk for *S. aureus* pneumonia.

## Methods

### Ethics statements

#### Human participants

*S. aureus* strains were obtained from adults 22–44 years of age in the CF program at Yale University, from children 0.5–2 years of age in the CF program at Seattle Children’s hospital and from a longitudinal collection from a single adult in the CF program at the University Hospital of Münster, all as part of routine care. None of the CF patients studied were under CF transmembrane conductance regulator modulator therapy (for example, Kaleydeco, Orkambi).

#### Bacterial strains

The bacterial strains used in this study are shown in Supplementary Table 4. All strains were grown at 37 °C on LB plates supplemented with 1% agar; LB broth shaking, with the exception of Newman c:*adsA* in/on LB + 10 mg l^−1^ chloramphenicol; USA300 c:*adsA* in/on LB + 5 μg ml^−1^ tetracycline; or *ccpA*::Tn, *ccpE*::Tn, *putP*::Tn in/on LB + 5 μg ml^−1^ erythromycin (Nebraska Transposon Mutant Library). For the experiments requiring supplementation with ATP, AMP, adenosine, proline, collagen, glycine, hydroxyproline or ornithine, the medium was pH corrected to 7.0 before filter sterilization using 0.20 μm filters. Inoculum was estimated on the basis of an optical density of 600 nm (OD_600_) and verified by serial dilution plating.

#### Mouse experiments

CD57/BL6 and 129S6/SvEvTac mice 8–10 weeks old were purchased from Jackson Laboratories and Taconic, respectively, and housed in a humidity-controlled facility at 18–23 °C and 12 h light/dark cycles. All animal studies were approved (Columbia Institutional Animal Care and Use Committee Protocol AABE8600) and performed in accordance with the Guide for the Care and Use of Laboratory Animals of the National Institutes of Health (NIH), the Animal Welfare Act and US federal law. Two transgenic strains were utilized: GFP FoxP3 DTR (stock number 016958) with background (stock number 005304) and CD73^−/−^ (stock number 018986) with background (stock number 000664). Each in vivo and ex vivo experiment was performed using an equal ratio of male to female animals. Sex was not expected to influence results. Animals were randomly assigned to cages, and their health was routinely monitored by an Institutional Animal Care and Use Committee veterinarian.

### Methods details

#### Materials and resources

A comprehensive list of materials and resources has been provided in Supplementary Table 4.

#### Cloning of USA300 *adsA* mutant

The *adsA* mutant in strain background USA300 was generated using the pIMAY protocol^21,51^. A fully synthetic 1,200 bp double-stranded DNA fragment was designed and ordered from Invitrogen (GeneArt). This DNA fragment contained the following features (from 5′ to 3′): SphI and BamHI restriction sites, a Pxyl/tet promoter, a ribosome-binding site, a codon-optimized pheS* gene, and KpnI and BglII restriction sites. The synthetic DNA was cloned in the topoisomerase vector pCR8 and transformed into XL1-Blue cells, resulting in strain ANG3944. The resulting plasmid pCR8-pheS* was found to have two base exchanges in the 10 promoter region, altering the optimal sequence TATAAT to CTTAAT (Pxyl/tet*). The Pxyl/tet* pheS* construct was sub-cloned from pCR8-pheS* via the SphI and BglII restriction sites into pIMAY [7], and the resulting plasmid pIMAY* was introduced into XL1-Blue, creating strain ANG4005. pIMAY* was then shuttled through IM08B [18], yielding strain ANG5002, and further used to transform *S. aureus* RN4220 and LAC*, creating strains RN4220 pIMAY* (ANG5079) and LAC* pIMAY* (ANG5080), respectively.

Plasmid pIMAY*-DmgtE was constructed by amplifying 1,000 base pairs of genomic DNA upstream and downstream of mgtE from *S. aureus* LAC* (ANG1575) chromosomal DNA using primer pairs P2374 (TATA CCCGGG AATGT- TAATT CAATACAATA CCGTGTAGC)/P2375 (AAAT- GATGTA GCACGCTCTT TTTCATCTGT GTTCATTGAC) and P2376 (GAAAAAGAGC GTGCTA- CATC ATTTATGGCT TACTTAATTT AAG)/P2377 (ACGT GAATTC TTGAAATGAT AAATGCAACG ATTAAAATCG), which were then spliced together using the primer pair P2374/P2377. This fusion construct contained the first and last 30 bases of mgtE and thus created a non-functional truncated open reading frame with flanking regions. The spliced DNA was cut with XmaI and EcoRI, ligated into pIMAY* (ANG5002) cut with the same enzymes and introduced into XL1-Blue, yielding strain ANG5081. The plasmid was subsequently introduced into IM08B to create strain ANG5082, and the plasmid isolated from this strain was introduced into *S. aureus* strains RN4220 [36] and LAC*. Two colonies each were selected, yielding strains ANG5083–86, and used in the allelic exchange procedure.

For the construction of the complementation plasmid piTET-mgtE, the mgtE gene, including its ribosome-binding site, were amplified using primers P2384 (CGATCCTAGG ACAGGGGGTGTAAGTATGTCAATGAACAC) and P2385 (AAGCAGATCT TTAAATTAAGTAAGCCA TAAATGATGTAGC). The PCR product was cut with AvrII and BglII and ligated with plasmid piTET (ANG284), which had been cut with the same enzyme. The resulting plasmid was recovered in *Escherichia coli* XL1-Blue cells, yielding strain ANG4144. The plasmid was then shuttled through IM08B, creating strain ANG4155, and used to transform one of the LAC* DmgtE deletion strains (ANG5091), resulting in the construction of the complementation strain LAC*DmgtE piTET-mgtE (ANG5288). In addition, the empty vector piTET isolated from strain ANG3928 was introduced into the mgtE mutant strain LAC*DmgtE 581 (ANG5091), (ANG5287) creating strain LAC*DmgtE piTET.

#### RNA-sequencing of *S. aureus* Newman WT and Δ*adsA*

*S. aureus* WT and Δ*adsA* were grown in LB with or without 100 μM proline to late exponential phase. Bacterial pellets were incubated in a cell wall lysis mixture TE buffer at pH 8 (30 mM Tris, 1 mM EDTA, 15 mg ml^−1^ and 200 μg ml^−1^ proteinase K) containing mutanolysin, lysostaphin and lysozyme at 37 °C for 30 min. TRK lysis buffer Total RNA lysis Kit (Omega #PRQ21) and 70% ethanol were added to each sample, before transferring to E.Z.N.A RNA isolation columns. RNA was isolated following the manufacturer’s instructions, and DNA was selectively degraded using the DNA-free DNA removal kit. The RNA was precipitated with 0.1 volume 3 M sodium acetate (Thermo Fisher #S209) and 3 volumes of 100% ethanol, recovered by centrifugation and washed with ice-cold 70% ethanol. A ribosomal RNA-depleted complementary DNA library was prepared according to the manufacturer’s instructions using the Universal Prokaryotic RNA-Seq Prokaryotic AnyDeplete kit (NuGEN #0363-32) and sequenced with Illumina HiSeq. Raw base calls were converted to fatsq files using Bcl2fastqs. Filtered reads were aligned to the Newman (Refseq NC_009641.1) reference genome using STAR-Aligner v2.7.3a. The mapped reads were annotated for read groups and marked for duplicates using the Picard tools v2.22.3. The raw counts were quantified using Subreads:FeatureCounts v1.6.3 and processed for differential gene expression using DEseq2 in R v3.5.3. Analysis of sequencing for clinical isolates was performed by Windmüller and colleagues. Briefly, data were stratified into ≥2f, ≥4 f, ≥8 f and genes that were either not expressed in both isolates or not present on the genome level in both isolates. To exclude low abundant transcripts, a threshold of less than 3 reads per gene was used (accession number GSE268637).

#### Mouse lung infection

Mice were anaesthetized with a 20:1 ketamine xylazine formulation infected intranasally with 1 × 10^8^ colony-forming units (c.f.u.) of the specified *S. aureus* strain. Bacteria were delivered in 50 μl PBS, and sterile PBS was used as control (vehicle). All mice were killed 72 h post infection for BAL fluid collection and lung tissue processing. Lung tissue was homogenized through 40–70 μm cell strainers. Colony-forming units were determined by serially diluting aliquots of the BAL and of lung homogenates, which were plated on LB agar plates (with the exception of Newman c::*adsA* on LB + 10 mg l^−1^ chloramphenicol; USA300 c::*adsA* on LB + 5 μg ml^−1^ tetracycline; *ccpA*::Tn, *ccpE*::Tn and *putP*::Tn on LB + 5 μg ml^−1^ erythromycin). The remaining BAL was spun and stored for cytokine and metabolomic analysis. BAL and lung cells were treated with a hypotonic lysis solution for elimination of red blood cells, counted and prepared for fluorescence-activated cell sorting (FACS) as described below.

#### In vivo T_reg_ cell depletion

GFP FoxP3 DTR (strain number B6.129(Cg)-Foxp3tm3(DTR/GFP)Ayr/J; stock number 016958) allows for depletion of FoxP3^+^ cells upon daily administration of diphtheria toxin. Mice were injected intraperitoneally with 50 μg kg^−1^ diphtheria toxin resuspended in sterile water once a day for 5 days. Mice were inoculated on the third day and processed on the sixth day as indicated above.

#### Untargeted metabolomic analysis

Aliquots of BAL supernatants were mixed in equal volume (*v*/*v*) with methanol and stored at −80 °C. Metabolites were identified and quantified by high-resolution mass spectrometry. Sample runs were performed on a Q Exactive HF Hybrid Quadrupole-Orbitrap Mass Spectrometer (Thermo Fisher) coupled to a Vanquish UHPLC System (Thermo Fisher). Chromatographic separation was achieved on a Syncronis HILIC UHPLC column (2.1 mm × 100 mm × 1.7 µm, Thermo Fisher) using a binary solvent system at a flow rate of 600 µl min^−1^. Solvent A, 20 mM ammonium formate at pH 3.0 in mass spectrometry grade H_2_O; solvent B, mass spectrometry grade acetonitrile with 0.1% formic acid (%*v*/*v*). A sample injection volume of 2 μl was used. The mass spectrometer was run in negative full-scan mode at a resolution of 240,000 scanning from 50 to 750 *m*/*z*. Metabolites were identified using the known chromatographic retention times of standards, and metabolite signals were quantified using E-Maven v0.10.0.

#### Pathway analyses

Following bulk RNA sequencing, bacterial pathways were identified using the National Institutes of Allergy and Infectious Diseases (NIAID)/NIH free Database for Annotation, Visualization and Integrated Discovery. The use of the tool relies on the identification of the bacterial species and genus and specific gene coding. In brief, a comprehensive rubric of NCTC8325 codes in the SAOUHSC ortholog format matching the sequencing locus discovery was assembled manually using the AureoWiki free online repository unique to *S. aureus*. The input of the codes in Database for Annotation, Visualization and Integrated Discovery generated unbiased functional annotations and pathway suggestions as well as a list of corresponding gene counts per pathway.

Following untargeted metabolomics of BAL fluid, host pathways were identified using the Ingenuity Pathway Analysis bioinformatic software (QIAGEN). In brief, a rubric of the Kyoto Encyclopedia of Genes and Genomes metabolite codes matching the macromolecular discovery was assembled. Data were paired for *t*-Student comparison and averaged to a single value for the generation of a fold change (log_2_). The input of the above in Ingenuity Pathway Analysis generated unbiased pathway suggestions as well as significance with respect to cumulative pathway *P* values and relative fold change.

#### Cytokine analysis

Aliquots of BAL supernatants were stored at −80 °C. Cytokine concentrations in BAL supernatants were quantified with the Mouse Cytokine Proinflammatory Focused M31-plex Discovery Assay (Eve Technologies), which quantifies eotaxin, G-CSF, GM-CSF, IFNγ, IL-1α, IL-1β, IL-2, IL-3, IL-4, IL-5, IL-6, IL-7, IL-9, IL-10, IL-12p40, IL-12p70, IL-13, IL-15, IL-17A, IP-10, KC, LIF, LIX, MCP-1, M-CSF, MIG, MIP-1α, MIP-1β, MIP-2, RANTES, TNFα and VEGF-A; TGFβ 3-plex, which quantifies TGFβ1, TGFβ2 and TGFβ3; and MMP 5-plex, which quantifies MMP-2, MMP-3, MMP-8, proMMP-9 and MMP-12, using a bead-based multiplexing technology also known as addressable laser bead immunoassay.

#### FACS

BAL and lung cells were treated with a hypotonic lysis solution for elimination of red blood cells and stained with fluorescent antibodies as follows. All antibodies were purchased on BioLegend unless otherwise indicated—lymphocytes: live/dead, CD45, CD4, CD8, CD25, FoxP3, CD39 and CD73; granulocytes and leukocytes: SiglecF, CD11b, CD11c, MHCII, LY6G, LY6C, CD39 and CD73; fibroblasts: live/dead, CD45, Dump gate (CD31, EPCAM, TER119, THY 1.2, CD146), SCA-1 and CD140a. Gating strategies are shown in Supplementary Figure 4.

All antibodies were used at a dilution of 1:200 with the exception of CD45 and FoxP3 (1:100) and CD25 (1:50). All dilutions were performed in PBS, and cells were stained for 1 h at 4 °C. For lymphocytes, after washing, the cells were fixed, permeabilized and intracellularly stained with a monoclonal anti-FoxP3 antibody in permeabilization buffer for 30 min at room temperature. After a final wash, the cells were stored in 2% paraformaldehyde until analysis on the BD LSRII (BD Biosciences) using FACSDiva v9. Flow cytometry was analysed with FlowJo v10.

#### Primary fibroblast isolation

Immediately after euthanasia of the experimental animal, the chest cavity was opened and the lungs flushed with PBS through the heart from the right ventricle. The lungs were both inflated and immersed in dispase (~0.9 ml, 50 U ml^−1^), incubated at room temperature for 20 min and isolated from surrounding structures. Lobes were chopped mechanically and incubated at 37 °C for 10 min in MEM + DNase to complete digestion. The mixed cells were filtered through 100 µm and 40 µm cup filters, and fibroblasts were removed from the suspension by three successive adherence steps on plastic. With respect to other cells, fibroblast adhesion is faster and facilitated by the continuous and sequential generation of filopodial structures containing integrins^52^. Moreover, adhesion is facilitated by the treated plasma of cell culture plates, which permits obtaining a consistent cell attachment and growth. Primary fibroblasts are washed three times with 1× PBS and incubated with Dulbecco's modified Eagle's medium (DMEM) 10% fetal bovine serum (FBS) and antibiotics.

#### Primary murine fibroblast immunostaining and imaging

Primary fibroblasts were fixed with 4% paraformaldehyde for 10–20 min at room temperature and washed three times with PBS for 5 min. The cells were permeabilized with 0.25% Triton X-100/PBS for 30 min at room temperature followed by blocking in 10% donkey serum diluted in PBS for 1 h. Vimentin primary antibody was diluted (1:400) in 5% donkey serum in PBS and incubated at 4 °C overnight. The following day, cells were washed three times with PBS before applying the secondary antibody (1:400) for 1 h incubation at room temperature. Following secondary incubation, sections were washed three times for 10 min with PBS and finally stained with DAPI (1:1,000) for 10 min at room temperature. PenStrep (1%) was added in 200 μl PBS before imaging with a motorized Leica Stellaris DMi8 (Leica Microsystems) inverted confocal microscope. Positive cells were counted over total number of cells. LasX v1.4.5 was used for fibroblast imaging.

#### Primary murine fibroblast infection time course

Primary fibroblasts were isolated from mouse lungs as described above and incubated in antibiotics for 4–5 days until 70–80% confluency was achieved. Cells were counted and plated (including two extra wells per plate) the day before infection. On the morning of the infection, the two extra wells were counted to determine the multiplicity of infection (MOI). After media change, the cells were infected with bacterial subcultures of WT and Δ*adsA* Newman strains grown to late exponential phase at a MOI of 10, or treated with PBS control. The infection was allowed to proceed until pre-determined time points. Processing of plates at the selected time points of 2, 24 and 72 h involved retrieving and plating one aliquot of supernatant for c.f.u. counts to determine extracellular multiplication due to collagen consumption. After 1 h incubation at 37 °C in 500 ng ml^−1^ gentamicin, aliquots of supernatants were collected and stored at −80 °C for protein quantification and RNA isolation before addition of 0.25% trypsin for 9 min at 37 °C. Aliquots of cells were either stored at −80 °C or used to measure cell viability.

#### Inhibition of collagen synthesis

Halofuginone, a semisynthetic quinazolinone (Collgard Biopharmaceuticals), was reconstituted in DMSO at a stock concentration of 5 mg ml^−1^.

For in vitro study, inhibition of collagen synthesis in cell culture was achieved via the administration of 0.25 mg ml^−1^ of halofuginone per well^32^ once before infection.

For in vivo study, inhibition of collagen synthesis was achieved through the intraperitoneal administration of halofuginone at a dose of 0.1 mg kg^−1^ in 100 μl of di-water for 3 sequential days, starting from the day of infection^33,34^. The health of control/uninfected mice was monitored to survey potential effects of the drug or vehicle. Animal weight was recorded every 24 h until euthanasia.

#### Collagen detection enzyme-linked immunosorbent assay

Collagen was quantified using the Mouse Pro-Collagen alpha 1 enzyme-linked immunosorbent assay (ELISA) kit (abcam) according to manufacturer’s instructions. Briefly, supernatant and cell aliquots from in vitro primary fibroblast infections and from in vivo airway BAL fluid were treated solubilized with chilled 1× Cell Extraction Buffer PTR, incubated on ice for 20 min and centrifuged at 18,000 *g* for 20 min at 4 °C. Supernatants (50 μl) were transferred to the assay plate along with 50 μl of antibody cocktail (600 μl 10× capture antibody, 600 μl detector antibody and 2.4 ml antibody diluent 5BR) per well and incubated at 400 r.p.m. for 1 h at room temperature. After washing, 100 μl of tetramethylbenzidine (TMB) substrate was added, and the plate was incubated shaking for 10 min in the dark. The addition of 100 μl of stop solution occurred before the endpoint reading at OD 450 nm.

#### Collagen staining and pathology assessments

Following euthanasia, the mouse was tracheostomized and cannulated. The thorax was opened to facilitate lung expansion upon gentle infusion of formalin-free tissue fixative through the cannula, attached to a 5 ml syringe positioned above the mouse. Lungs were collected en bloc, placed in formalin-free tissue for 24 h, in 70% ethanol for 24 h and prepared in paraffin blocks. Haematoxylin and eosin and Masson’s trichrome staining were performed on 5 μm sections (two sections cut 25 μm apart for each lung) for gross pathology assessment.

#### In vitro RNA isolation

Bacterial strains Newman WT, Newman Δ*adsA* and all clinical isolates were grown to the exponential phase and normalized to an OD_600_ of 1. Bacteria were incubated in RNAprotect cell reagent and TE buffer at pH 8 (30 mM Tris, 1 mM EDTA and 200 μg ml^−1^ proteinase K) containing mutanolysin, lysostaphin and lysozyme at 37 °C for 30 min. TRK lysis buffer and 70% ethanol were added to each sample, before transferring to E.Z.N.A RNA isolation columns. RNA was isolated following the manufacturer’s instructions, and DNA was selectively degraded using the DNA-free DNA removal kit.

#### In vivo RNA isolation from mouse lungs

Total RNA (eukaryotic and prokaryotic) was isolated from infected whole mouse lungs added to TRIzol Reagent following the manufacturer’s instructions and homogenized. Aliquots of these samples were stored for immediate reverse transcription to obtain largely eukaryotic cDNA analysis. The remaining sample was depleted of eukaryotic RNA with a MICROBEnrich Kit. The samples were added to binding buffer and capture oligonucleotide mix, incubated at 70 °C for 10 min and 37 °C for 1 h to hybridize prokaryotic RNA with the oligonucleotides. Host RNA was then depleted using OligoMag beads, and enriched prokaryotic RNA was recovered following the manufacturer’s instructions. DNA was selectively degraded using the DNA-free DNA removal kit. Prokaryotic RNA was precipitated using MICROBEnrich Kit as per manufacturer’s instructions.

#### cDNA synthesis and quantitative real-time PCR

RNA was converted to cDNA using a High-Capacity cDNA Reverse transcription kit and a SimpliAmp thermocycler (Applied Biosystems). Quantitative real-time PCR was performed using the relevant primers and PowerUp SYBR Green PCR Mastermix. All primers used in this study are listed in Supplementary Table 1. The experiment was performed on a StepOnePlus Real-time PCR System (Applied Biosystems) using StepOne v2.3. Data were analysed using the ∆∆C_T_ method. 16S was used as a control housekeeping gene for bacteria, and the Newman strain of *S. aureus* was included as a wild-type control. GAPDH was used as a control housekeeping gene, and the uninfected hosts were used as controls.

#### Carbon utilization assays

*S. aureus* WT, *adsA* mutants and clinical isolates were grown to the exponential phase and normalized to an OD_600_ of 2. A stock solution of 2 × 10^7^ bacteria per ml was made in 1× IF-Oa buffer supplemented with 1× Redox Dye Mix H. About 100 μl of this stock solution was added to each well of a BIOLOG PM1 Microplate (BIOLOG) delivering 2 × 10^6^ bacteria. This plate was incubated at 37 °C for 24 h, and absorbance was read at 590 nm on a SpectraMax M2 plate reader.

#### Growth curves

A U-bottomed, clear 96-well plate was prepared with LB or chemically defined media (CDM), LB or CDM supplemented with 100 µM proline and LB or CDM supplemented with 30–10 µg ml^−1^ collagen. Each well was inoculated with 2 × 10^6^ bacteria. Absorbance at 600 nm was read every 30 min for 20–50 h on a SpectraMax M2 plate reader. The plate was incubated at 37 °C with shaking before and after every read. Compound are listed in Extended Data Table 5.

#### Biofilm assays

A flat-bottomed, clear, 96-well plate was prepared with LB or LB with 0.5% glucose (*w*/*v*), supplemented with serially diluted, pH-corrected proline. Each well was inoculated with 1.5 × 10^6^ bacteria, and the plate was left to incubate statically overnight at 37 °C. The next morning, absorbance at 600 nm was determined on an Infinite M200 plate reader (Tecan). To stain the biofilm, the supernatant was discarded, the plate was washed and dried, and the biofilm was fixed with 100% methanol, then stained with 1% crystal violet. After discarding the staining solution and washing and drying the plate, the stained biofilm was resuspended in 33% acetic acid. Absorbance at 540 nm was determined on the Infinite M200 plate reader using iControl v1.10.4.

#### ATP luciferase assays

Bacterial strains were cultured overnight in LB + 100 μM proline or LB alone, standardized to an OD of 1 and subcultured 1:100 with the respective fresh broths for 2 h. In a 96-well plate, an ATP 10-fold serial dilution was used as internal standard control. ATP concentrations were measured as per BacTiter Glo Microbial Cell Viability Assay kit instructions. For extracellular ATP, supernatant was obtained from 100 μl of bacterial culture and mixed with assay reagents for 5 min before reading luminescence on a SpectraMax M2 plate reader. For intracellular ATP, 200 μl of bacterial culture was mixed with 100 μl of ice-cold 1.2 M perchloric acid, incubated on ice for 15 min, and neutralized with 0.72 M KOH and 0.16 M KHCO_3_.

#### Extracellular flux analysis

Bacterial cultures were grown overnight and subcultured. The XFe24 sensor cartridge was calibrated as per the manufacturer’s instructions overnight at 37 °C without CO_2_. About 500 μl of XF base medium supplemented with 2 mM glutamine was added to each well of a Seahorse XF24 well plate and inoculated with 2 × 10^6^ bacteria for a 1 h incubation at 37 °C. The OCR and ECAR were measured on a Seahorse XFe24 Analyzer (Agilent Technologies) using Seahorse Wave Desktop v2.6.0. Proline was added at a final concentration of 100 μM at the three time points indicated.

#### In silico bioinformatic identification of *ccpA* and *ccpE* binding motifs

De novo assembly of the sequenced airway isolates, A2001 and T2015, was performed using Shovill (v. 1.1.0) (https://github.com/tseemann/shovill). Following the completion of assembly, the genomes were identified as *S. aureus* using Mash (v. 2.1)^53^. Assembled genomes were annotated using Prokka (v.1.14.6)^54^. The atopic dermatitis genomes used in comparison—AD2 (GCF_011319675.1) and AD8 (GCF_011319565.1)—were downloaded from RefSeq database in the National Center for Biotechnology Information (NCBI) using ncbi-datasets (v. 14.6.0)^55^. Basic Local Alignment Search Tool (Blastn) (v. 2.13.0) was then used to confirm the presence of the genes of interest in each of these five genomes^56–58^. After confirming the presence of each of these genes, samtools (v. 1.16.1) was used to extract each gene^59^. In extracting each gene, 1,000 base pairs upstream from the start of the gene and 1,000 base pairs downstream from the end of the gene were included. Using a custom script, the extracted regions for each genome were evaluated for matches to the cis-acting sequence called catabolite responsive element (*cre*) sites. Taking into consideration that diverse *cre* sites have been published^39,60–62^, we used two candidate motifs and allowed up to two mismatches. One of the *cre* motifs that was searched for was WTGNAANCGNWNNCW, where W represents A or T, and N represents any nucleotide^44^. The other candidate sequence that was used in the custom script was TGTAAA-Yx-TTTACA, where N represents any base and ranges between 0 and 40 nucleotides^45^. The custom script reported the sequence that was identified, the motif that the sequence matched, the location of the sequence in relation to the start of the gene of interest and the number of substitutions (that is, 1 substitution, 2 substitutions or exact match). The best match was to be the sequence closest to the start of the gene of interest, with the least number of mismatches. We then checked for nonsynonymous mutations in *ccpA* and *ccpE*, in the four clinical isolates. A protein alignment was performed using Snippy (v. 4.6.0) (https://github.com/tseemann/snippy). The gene sequences of *ccpA* (region 1813146.1814135) and *ccpE* (region 724466.725332) from strain Newman (Refseq NC_009641.1) were used as the reference for each alignment. SnpEff (v. 4.3.0) was then used to analyse the effect of each single-nucleotide polymorphism on the protein sequence^63^. The alignments were visualized using snipit (v. 1.1.2), with the respective gene from strain Newman as the reference (https://github.com/aineniamh/snipit).

#### Bacterial luciferase reporter plasmid

To construct pIMK1-LUX, the *Listeria* phage integrase vector pIMK^64^ was digested with SphI/BglII to excise the PSA integrase and replace it with the PCR-amplified (IM1241/IM1242) low copy number pSK41 replicon from pLOW^65^, yielding pIMK1. Vectors pIMK1 and pPL2*lux* were then digested with SalI/PstI, and the gel-extracted pIMK1 backbone ligated to the bacterial luciferase operon from pPL2*lux*. The vector pIMK1-LUX produces exact promoter fusions which can be cloned into the SalI/SwaI digested vector, as described previously^64^. Promoters for *adsA* (IM1793/IM1794), *citB* (IM1785/IM1786) and *pckA* (IM1787/IM1788) were amplified from *S. aureus* strain JE2 genomic DNA. The amplimers were digested with SalI, gel extracted and cloned into the pIMK1-LUX double digested vector. Plasmids isolated from IM08B^66^ were transformed into *S. aureus* JE2, JE2 *ccpA*::Tn or JE2 *ccpE*::Tn and selected on brain heart infusion agar containing 50 µg ml^−1^ kanamycin. An overnight 5 ml LB culture containing kanamycin was diluted 1:100 in fresh LB containing kanamycin. The culture was then dispensed in triplicate (200 µl) black/clear bottom 96-well plates (PhenoPlate 96-well microplate, Perkinelmer). The plates were sealed with MicroAmp Optical Adhesive Film (Thermo Fisher) and incubated at 37 °C with dual orbital shaking at 300 r.p.m. (Clariostar Plus, BMG). Every 10 min the plate was read at OD_600_, and light emission (1 s exposure) collected over an 8 h period. Primers are listed in Supplementary Table 1.

#### Colony PCR

Transposon mutants from the Nebraska Transposon Mutant Library were confirmed via colony PCR. Primers were generated manually and consisted of the first and last 20 bp of the genes of interest. Agarose gels (1%) in 1× Tris–acetate–EDTA buffer were cast after addition of ethidium bromide and allowed to solidify. LB agar + erythromycin plates were streaked with mutants of interest (*ccpA*::Tn, *ccpE*::Tn, *putP*::Tn) and grown overnight. A minimum of 10 single colonies were picked per transposon mutant, and a minimum of 3 for the isogenic WT JE2, both to be resuspended in 25 μl NON-DEPC nuclease-free water. A Go-Taq master mix is prepared (12.5 μl Go Taq, 1 μl forward primer, 1 μl reverse primer, 5 μl emulsified colony, 5.5 μl nuclease-free water) and set at an enzyme/oligonucleotide-dependent thermal cycle (Applied Biosystems) as indicated in the Extended Data. Upon the completion of the cycle, the samples were loaded in the solidified agar gel soaked in an electrophoresis chamber with 1× Tris–acetate–EDTA at room temperature. Gel was run at 140 V for 20 min and imaged with a chemiluminescent detection imager (ProteinSimple). Emulsified colonies of confirmed mutants were streaked and incubated overnight.

#### In vivo skin infection

Mice were infected intradermally on the shaved back with 1 × 10^7^ c.f.u. of *S. aureus* Newman WT or *adsA* mutant. Bacteria were delivered in 100 μl PBS, and sterile PBS was used for an uninfected control (vehicle). Lesion size was measured daily, and mice were killed after 6 days for tissue collection and processing. Skin tissue was collected via 5 mm punch biopsy in the centre of the infected lesion and homogenized through a 40 μm cell strainer. Aliquots of the homogenates were serially diluted and plated on LB agar (±antibiotics if needed) plates to determine c.f.u. counts. Separate aliquots were added to TRK lysis buffer and frozen for later host RNA isolation.

#### Cloning of the Δ*adsA*Δ*putP*Δ*proT* USA300 mutant

The *putP* and *proT* genes were deleted (between the start and stop codon) from *S. aureus* LAC or LACΔ*adsA* by allelic exchange, as described in ref. ^67^. For the deletion substrate, gBlocks for *putP* (screening primers IM1789/IM1790) and *proT* (screening primers IM1791/IM1792) were synthesized by Integrated DNA Technologies, cloned into pIMAY-Z by SLiCE and transformed into *E. coli* IM08B. The genes were deleted sequentially, yielding LACΔ*proT*Δ*putP* and LAC Δ*adsA*Δ*proT*Δ*putP* and genome validated by Oxford nanopore sequencing^68^.

#### BMDM killing assay

Bone marrow was isolated from mouse femurs and tibias spun at 500 *g* for 6 min and resuspended in ammonium chloride potassium (ACK) lysis buffer for 5 min at room temperature. After lysis buffer inactivation, the cells were plated and differentiated in DMEM with penicillin–streptomycin in the presence of 20 ng ml^−1^ of murine macrophage colony stimulating factor (M-CSF) for 5 to 6 days (Peprotech). Newly differentiated BMDMs were seeded at 0.3 × 10^6^ cells per ml in DMEM supplemented with 1% FBS. BMDMs were infected at an MOI of 10 for 60 min and washed. Fresh DMEM containing 1% FBS and 50 μg ml^−1^ gentamicin (Sigma) was then added until the desired time point. The cells were washed, detached using TrypLE Express (Life Technologies), serially diluted and plated on LB agar at 2, 4 and 6 h post infection. Cell viability was determined by trypan blue (Life Technologies) staining assay.

### Quantification and statistical analysis

Experiments in this study were not performed in a blinded fashion except for the histopathology qualitative score assessments. Animal assignment to control or experimental groups was randomized. No statistical methods were used to pre-determine sample sizes, but our sample sizes are similar to those reported in previous publications from our laboratory. All analyses and graphs were performed using the GraphPad Prism 9 software. Values defined as *n* represent individual experiments, and graphed data points represent total biological replicates (please note that some replicates with value 0 will not be visible on logarithmic scale). Values in graphs are shown as average ± s.e.m., and data were assumed to follow a normal distribution. For comparison between average values for more than two groups, we performed one-way analysis of variance (ANOVA) with a multiple posteriori comparison (Tukey’s multiple comparisons or Dunnett’s multiple comparisons). When studying two or more groups along time, data were analysed using two-way ANOVA with a multiple posteriori comparison. Differences between two groups were analysed using a Student *t*-test with Kolmogorov–Smirnov test. Differences were considered significant when at least a *P* value under 0.05 (*P < 0.05) was obtained (**P < 0.01; ***P < 0.001; ****P < 0.0001). Statistical details of experiments are indicated in each figure legend. Data distribution was assumed to be normal. No data points were excluded.

### Reporting summary

Further information on research design is available in the Nature Portfolio Reporting Summary linked to this article.

## Supplementary information

**Extended Data Fig. 6:**
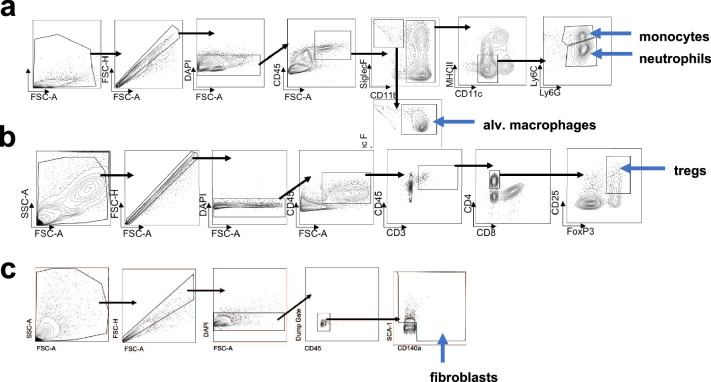
FACS Gating strategies. **a**, Neutrophils, alveolar macrophages and monocytes; **b**, lymphocytes and T regulatory cells; and **c**, lung fibroblasts.

Supplementary Information Legends for Extended Data Figs. 1–6 and Supplementary Tables 1–3.

Reporting Summary

Supplementary Tables Supplementary Tables 1–6.

## Data Availability

The authors confirm that the data supporting the sequencing findings in this study are available at GEO (accession number GSE268637), Zenodo (10.5281/zenodo.11450126) (ref. ^69^) and NCBI (Bio Project PRJNA1115226, accession numbers JBECZQ000000000 and JBECZR000000000). Bacterial strains used in this study are available from the lead contact without restriction. Data, material and resources requests should be directed to and will be fulfilled by the lead contact, A.S.P.

## Code availability

No custom code was used or developed during the analysis of the data in this study.
