# Supplementary material for: Staphylococcus aureus adapts to exploit collagen-derived proline during chronic infection
Source: Nat Microbiol. 2024 Aug 12;9(10):2506–21. doi: 10.1038/s41564-024-01769-9 (PMC11445067; doi:10.1038/s41564-024-01769-9)
Supplement: Supplementary file 1 — Legends for Extended Data Figs. 1–6 and Supplementary Tables 1–3. [file 41564_2024_1769_MOESM1_ESM.pdf]

nature microbiology

Supplementary information

https://doi.org/10.1038/s41564-024-01769-9

Staphylococcus aureus adapts to exploit collagen-derived proline during chronic infection

In the format provided by the authors and unedited

Extended Data Figure Legends

Figure S1. Relative single carbon source consumption by *S. aureus* strains. a, Utilization of carbon sources by CF clinical isolates (2001, 2015, CF1, CF9) and Newman *adsA* mutants shown as fold-change respective to Newman WT. **b,** Growth of *S. aureus* WT Newman and Δ*adsA* in CDM **b,** ± 100μM hydroxyproline (HyP), **c,** ± 100μM glycine (G), and c:adsA in **d,** CDM and **e,** CDM + proline. **f, g,** Newman and LAC plasmid stability in LB ± antibiotics. Mean ± SEM, n=3. Significance determined by **P* < 0.05; ***P* < 0.01; ****P* < 0.001 *****P* < 0.0001, (**a**) two-tailed t-Student with Kolmogorov-Smirnov test; (**b-e**) One-Way ANOVA with Tukey's Multiple Comparisons; (**f, g**) Two-Way ANOVA with Dunnett's Multiple Comparisons.

Figure S2. Impact of host generated adenosine on WT and Δ*adsA* mutant infection. Pulmonary infection at 72 hours following intranasal inoculation with *S. aureus* WT Newman, Δ*adsA* or PBS. **a,** Untargeted metabolomics shown respective to PBS control, **b,** T regulatory cell (Tregs) count and **c,** relative expression of surface CD39 and CD73 ectonucleotidases on various immune cells. Pulmonary infection at 72 hours following intranasal inoculation of GFP FoxP3^{DTR} mice with *S. aureus* WT Newman or Δ*adsA* and *in vivo* diphtheria toxin (DT)-mediated Treg depletion. **d,** Total lung Tregs **e,** bacterial burden **f,** immune cell populations and **g,** CD39 and CD73 expression upon treatment with DT or vehicle. Pulmonary infection at 72 hours following intranasal inoculation of CD73-KO mice with *S. aureus* WT Newman or Δ*adsA.* **h,** Bacterial burden in WT and CD73-KO mouse lungs. **i,** Weight loss, **j-o,** lung immune cells, and **p,** untargeted metabolomics on BAL fluid respective to PBS. Mean ± SEM, (**a, b**) n=3; (**c-e, h-p**) n=3; (**f**) n=4. Significance determined by **P* < 0.05; ***P* < 0.01; ****P* < 0.001 *****P* < 0.0001; (**a-h. j-p**) One-Way ANOVA with Tukey's Multiple Comparisons; (**f**) Two-Way ANOVA with Dunnett's Multiple Comparisons.

Figure S3. Consumption of components of the citrulline superpathway by CF clinical isolates. Growth of **a-e,** WT, Δ*adsA,* **f-j,** A2001 and T2015 in **a, f,** complete

defined medium (CDM) lacking **b, g,** proline, **c, h,** histidine, **d, i,** glutamate or **e, j,** arginine. Mean ± SEM from (**a-j**) n=4.

Figure S4. Induction of collagen synthesis by WT and *ads*A infection. Induction of host responses by pulmonary infection with *S. aureus* WT or Δ*adsA* strains at 72h. **a,** BAL fluid matrix metalloprotease and **b,** pro-inflammatory cytokines quantified by ELISA and plotted with respect to PBS. Primary murine fibroblasts were freshly isolated and cultured for infection with *S. aureus* WT or Δ*adsA* strains and treatment with collagen inhibitor halofuginone (HF) ex vivo. **c,** Representative lung sections stained with Masson's Trichrome (purple) and hematoxylin and eosin staining (pink) from a PBS control, WT Newman and Δ*adsA* infection at 72 h are shown. Scale bar= 50μM; m=40x. **d,** Representative confocal images of primary fibroblasts (PF) stained with vimentin and DAPI to confirm population purity (scale bar 40μm) . **e,** Cell count of live PF with trypan blue at each infection timepoint *in vitro*. *S. aureus* growth in **f, g**, LB only and **h, i,** CDM+P supplemented with HF or vehicle. *In vivo* intranasal 72-hour lung infection with *S. aureus* WT or Δ*adsA* strains in mice treated with HF or vehicle. **j,** BAL and **k,** lung total cells from infected mice treated with HF or vehicle. Mean ± SEM from (**a-b**) n=3; (**e**) n=4; (**f-i**) n=6; (**j-k**) n= 6. (**a-b, d-j**) Two-Way ANOVA with Dunnet's Multiple Comparison.

Figure S5. Impact of *adsA* and CCR on skin infection. **a**, RNA transcripts from *S. aureus* Atopic Dermatitis (AD) clinical isolates. **b**, Carbon utilization assays shown as fold-change increase w.r.t. WT. **c**, Growth curves in CDM ± 100μM proline. **d**, Bacterial burden and **e**, skin lesion size at 6 days post-infection from a 5mm punch biopsy of skin infected with *S. aureus* Newman. **f**, Host RNA transcripts from 5mm punch biopsy of skin infected with *S. aureus* Newman. **g-h**, Putative *cre* binding sites in *S. aureus* atopic dermatitis (AD) isolates AD2 and AD8 for *ccpA* in silico (black dashes, mismatches; red dots, matches) using binding sequences Sequence 1 and Sequence 2 (red). **i**, Bacterial burden at 6 days post-infection from a 5mm punch biopsy of skin infected with WT JE2, *ccpA::*Tn, *ccpE::*Tn, *putP::*Tn. Data presented as mean ± SEM, (**a-c**) *n*=3, (**d,e**) *n*=4, (**i**) *n=3*. Significance determined by **P* < 0.05, ***P* < 0.01, ****P* < 0.001, *****P* < 0.0001, (**a,c,e**)

Two-Way ANOVA with Dunnett's Multiple Comparisons, (**b,d**) two-tailed t-Student with Kolmogrov-Smirnov test (**f,i**) One-Way ANOVA with Tukey's Multiple Comparisons.

Figure S6. FACS Gating strategies for **a,** neutrophils, alveolar macrophages and monocytes; **b,** lymphocytes and T regulatory cells; and **c,** lung fibroblasts.

<u>Supplemental Tables Legends</u>

Table S1. Primers used in this study.

Table S2. CF clinical isolate genomic polymorphisms. Isolates 2001 and 2015 were sequenced and compared to *S. aureus* Newman WT strain (Accession NC_009641.1) **a,** synonymous and non-synonymous mutations and **b,** polymorphisms in genes of interest. *SUB= substitution, *EM= exact match

Table S3. CcpE CRE binding site matches in representative CF clinical isolates. Sequenced genomes of isolates 2001 and 2015 were surveyed for CRE binding sequence (ATAANNNNNNTTAT) for CcpE in promoter and open reading frame regions of genes of interest. *SUB= substitution, *EM= exact match.
