# Supplementary material for: Staphylococcus aureus adapts to exploit collagen-derived proline during chronic infection
Source: Nat Microbiol. 2024 Aug 12;9(10):2506–21. doi: 10.1038/s41564-024-01769-9 (PMC11445067; doi:10.1038/s41564-024-01769-9)
Supplement: Supplementary file 2 — Reporting Summary [file 41564_2024_1769_MOESM2_ESM.pdf]

nature portfolio

Corresponding author(s): Alice S. Prince MD

Last updated by author(s): 03/26/2024

Reporting Summary

Nature Portfolio wishes to improve the reproducibility of the work that we publish. This form provides structure for consistency and transparency in reporting. For further information on Nature Portfolio policies, see our Editorial Policies and the Editorial Policy Checklist.

Statistics

For all statistical analyses, confirm that the following items are present in the figure legend, table legend, main text, or Methods section.

n/a	Confirmed	
☐	☒	The exact sample size (*n*) for each experimental group/condition, given as a discrete number and unit of measurement
☐	☒	A statement on whether measurements were taken from distinct samples or whether the same sample was measured repeatedly
☐	☒	The statistical test(s) used AND whether they are one- or two-sided<br>*Only common tests should be described solely by name; describe more complex techniques in the Methods section.*
☒	☐	A description of all covariates tested
☐	☒	A description of any assumptions or corrections, such as tests of normality and adjustment for multiple comparisons
☐	☒	A full description of the statistical parameters including central tendency (e.g. means) or other basic estimates (e.g. regression coefficient) AND variation (e.g. standard deviation) or associated estimates of uncertainty (e.g. confidence intervals)
☐	☒	For null hypothesis testing, the test statistic (e.g. *F*, *t*, *r*) with confidence intervals, effect sizes, degrees of freedom and *P* value noted<br>*Give P values as exact values whenever suitable.*
☒	☐	For Bayesian analysis, information on the choice of priors and Markov chain Monte Carlo settings
☒	☐	For hierarchical and complex designs, identification of the appropriate level for tests and full reporting of outcomes
☒	☐	Estimates of effect sizes (e.g. Cohen's *d*, Pearson's *r*), indicating how they were calculated

Our web collection on statistics for biologists contains articles on many of the points above.

Software and code

Policy information about availability of computer code

Data collection	BD FACSDiva v9 was used for acquisition of the flow cytometry data. Seahorse Wave Desktop v2.6.0 was used for the acquisition of the extracellular flux data. StepOne v2.3 was used for acquisition of the qRT-PCR data. Tecan iControl v1.10.4 was used for the acquisition of the growth curves, ATP and carbon source assimilation data. Q Exactive™ HF Hybrid Quadrupole-Orbitrap™ Mass Spectrometer (Thermo-Fisher) coupled to a Vanquish™ UHPLC System (Thermo-Fisher), with software E-MAVEN v0.10.0 and MAVEN 2011.6.17 were used for acquisition of metabolomics data. Ingenuity Pathway Analysis (IPA) bioinformatic software (QIAGEN) was used for pathway analysis. Leica Stellaris DMi8 (Leica Microsystems, Buffalo Grove, IL) inverted confocal microscope was used to collect immunofluorescence imaging of fibroblasts. Accuris instruments Smartreader 96 #MR9600 Version a1.1.3 210610 was used for ELISA. Illumina HiSeq platform was used to acquire the RNA sequencing data.
Data analysis	E-MAVEN v0.10.0 and MAVEN 2011.6.17 were used to quantify metabolite signals. FlowJo v10 was used for cell gating and signal quantification. Shovill v 1.1.0, Mash v2.1, Prokka v1.14.6, Blatn v2.13.0, Samtools v1.16.1 were used to align, annotate, confirm the bacterial genome and extract the genes of interest, respectively. Snippy v4.6.0, SnpEff v4.3.0, snipit v1.1.2 were used for protein alignment, SNP effect prediction and visualization. Seahorse Wave Desktop v2.6.0 was used to analyze extracellular acidification rates and oxygen consumption rates. LasX v1.4.5 was used for fibroblast imaging. Prism v9 was used to analyze and plot collected data. STAR-Aligner v2.7.3a, Picard tools v2.22.3, Subreads:FeautreCounts v1.6.3 and DEseq2 in R v3.5.3 were used to read, map, mark and quantify the differential gene expression in RNA sequencing data. Database for Annotation Visualization and Integrated Discovery (DAVID) was used to analyze RNA-seq data.

For manuscripts utilizing custom algorithms or software that are central to the research but not yet described in published literature, software must be made available to editors and reviewers. We strongly encourage code deposition in a community repository (e.g. GitHub). See the Nature Portfolio guidelines for submitting code & software for further information.

Data

Policy information about availability of data

All manuscripts must include a data availability statement. This statement should provide the following information, where applicable:

- Accession codes, unique identifiers, or web links for publicly available datasets
- A description of any restrictions on data availability
- For clinical datasets or third party data, please ensure that the statement adheres to our policy

Sequencing data is publicly available and can be accessed on GEO via the accession code _____.Requests for data, materials and resources should be directed to and will be fulfilled by the lead contact, Alice Prince (asp7@cumc.columbia.edu).

Research involving human participants, their data, or biological material

Policy information about studies with human participants or human data. See also policy information about sex, gender (identity/presentation), and sexual orientation and race, ethnicity and racism.

Reporting on sex and gender	N/A
Reporting on race, ethnicity, or other socially relevant groupings	N/A
Population characteristics	N/A
Recruitment	N/A
Ethics oversight	N/A

Note that full information on the approval of the study protocol must also be provided in the manuscript.

Field-specific reporting

Please select the one below that is the best fit for your research. If you are not sure, read the appropriate sections before making your selection.

☒ Life sciences ☐ Behavioural & social sciences ☐ Ecological, evolutionary & environmental sciences

For a reference copy of the document with all sections, see nature.com/documents/nr-reporting-summary-flat.pdf

Life sciences study design

All studies must disclose on these points even when the disclosure is negative.

Sample size	Samples sized were determined by power analysis based on the effect of sizes in previous experiments: power of 0.8 and significance of 0.05 for statistical tests.
Data exclusions	No data points were excluded.
Replication	To ensure the reproducibility of the data, both technical replicates and biological replicates were used. Biological replicates were derived from biologically independent mice, cell cultures, and/or experiments (performed on different days, with different samples and controls). The details for how often independent experiments were performed for are provided in the figure legends. For each experiment, all attempts at replication were successful.
Randomization	Samples and animals were randomly allocated into experimental groups.
Blinding	Blind assessments were performed for the histopathological evaluation of slides only.

Reporting for specific materials, systems and methods

We require information from authors about some types of materials, experimental systems and methods used in many studies. Here, indicate whether each material, system or method listed is relevant to your study. If you are not sure if a list item applies to your research, read the appropriate section before selecting a response.

Materials & experimental systems

n/a	Involved in the study	
☐	☒	Antibodies
☐	☒	Eukaryotic cell lines
☒	☐	Palaeontology and archaeology
☐	☒	Animals and other organisms
☒	☐	Clinical data
☒	☐	Dual use research of concern
☒	☐	Plants

Methods

n/a	Involved in the study	
☒	☐	ChIP-seq
☐	☒	Flow cytometry
☒	☐	MRI-based neuroimaging

Antibodies

Antibodies used	Antibodies used in this study include: anti-CD45-AF700 (BioLegend cat# 103127), anti-CD11b-AF594 (BioLegend cat# 101254), anti-CD11c-Bv605 (BioLegend cat# 117334), anti-SiglecF-PE (BD Biosciences cat# 552126), anti-Epcam-FITC (BioLegend #118207), MHCII - APC-Cy7 (BioLegend cat#107628), anti-Ly6C- FITC (BioLegend cat# 128032), anti-Ly6G-PerCp-Cy5.5 (BioLegend cat# 127616), CD3-AF594 (BioLegend cat# 100240), CD4-Percp-Cy5.5 (BioLegend cat#100540), CD8-AF488 (BioLegend cat#100706), CD25-Bv510 (BioLegend cat#102041), FoxP3-APC-Cy7 (Invitrogen cat#25-5773-82), CD39-APC (BioLegend cat#143810), CD73-Bv421 (BioLegend cat#127217), CD31-FITC (BioLegend cat#102506), TER119-FITC (BioLegend cat#116205), THY1.2-FITC (BioLegend cat#105305), CD146-FITC (BioLegend cat#134705), SCA-1-APC-Cy7 (BioLegend cat#108125), CD140a-PE (BioLegend cat#135905), CD103-Bv510 (BioLegend cat#121423),Anti-rabbit IgG HRP-linked (Cell Signaling, cat#7074S), His-Tag Rabbit (Cell Signaling, cat#2365S).
Validation	Target specificity and functional validation for each antibody was performed by the manufacturer, and validation statements for each antibody can be found on the manufacturer's website as approved for this use. All antibodies were tested before use in these experiments.

Eukaryotic cell lines

Policy information about cell lines and Sex and Gender in Research

Cell line source(s)	The 3T3 NIH cell line was sourced from co-authors Nicolino Valerio Dorrello and Camilla Predella as indicated in the methods and acknowledgments and has been expanded to working stocks within our lab.
Authentication	The 3T3 NIH cell line was authenticated by the supplier and were authenticated by morphology. These cells only served as comparative elements for extracted primary fibroblasts.
Mycoplasma contamination	The cell lines were not tested for mycoplasma contamination.
Commonly misidentified lines (See ICLAC register)	None.

Animals and other research organisms

Policy information about studies involving animals; ARRIVE guidelines recommended for reporting animal research, and Sex and Gender in Research

Laboratory animals	In this study, 8-10 week-old, mixed-sex mice (WT C57Bl6N, stock number 000664, from Jackson Laboratories) were used. These mice were housed in humidity-controlled conditions at 18-23 degrees Celsius, with 12 hour light/dark cycles.
Wild animals	This study did not involve wild animals.
Reporting on sex	Both sexes were used in this study. Findings apply to both sexes.
Field-collected samples	This study did not involve field-collected samples.
Ethics oversight	All animal studies were subject to oversight by the Columbia Institutional Animal Care and Use Committee (IACUC) and were approved under protocol AABE8600.

Note that full information on the approval of the study protocol must also be provided in the manuscript.

Flow Cytometry

Plots

Confirm that:

☒ The axis labels state the marker and fluorochrome used (e.g. CD4-FITC).

☒ The axis scales are clearly visible. Include numbers along axes only for bottom left plot of group (a 'group' is an analysis of identical markers).

☒ All plots are contour plots with outliers or pseudocolor plots.

☒ A numerical value for number of cells or percentage (with statistics) is provided.

Methodology

Sample preparation	For the cells harvested from infected mice: bronchoalveolar lavage fluid (BAL) was collected by intratracheal lavage and lung tissue was collected and homogenized through 40 um cell strainers. Red blood cells were lysed hypotonically in ACK lysis buffer, and the remaining cells were extracelullarly stained in buffered saline, fixed, permeabilized, and intracellularly stained using the eBiosceince FoxP3/transcription factor staining buffer set, and stored in 2% paraformaldehyde until analysis.
Instrument	BD LSRII was used to for the analysis.
Software	BD FACSDiva v9 was used for data acquisition and FlowJo v10 was used for data analysis.
Cell population abundance	No post-sort fractions were collected in this study.
Gating strategy	For the infected mouse BAL and lung samples: cells were gated using forward scatter and side scatter, single cells were gated using forward scatter (area and height), live cells were gated using forward scatter and DAPI, and CD45+ (vs. forward scatter area) immune cells were gated using AF700; within that population SiglecF+CD11b-CD11c+ alveolar macrophages using PE, AF594, Bv605; CD11b+MHCII-CD11c- cells were further gated into Ly6C+/Ly6G- monocytes and Ly6G+ Ly6C+/- neutrophils, using APC-Cy7 for MHCII, FITC and Percp-Cy5.5 for Ly6C and Ly6G respectively . From the CD45+ cells, CD3+ cells were gated using AF594 and subdivided into CD4+ and CD8+ using PercP-Cy5.5 and AF488 respectively. CD4+ cells were further gated into CD25+ FoxP3+ T regulatory cells using Bv510 and APC-Cy7. Fibroblasts were isolated with the use of a dump gate inclusive of CD45-CD31-EPCAM-TER119-THY1.2-,CD146- using FITC, which were then further gated into SCA-1+ CD140a+ cells using APC-Cy7 and PE.

☒ Tick this box to confirm that a figure exemplifying the gating strategy is provided in the Supplementary Information.
